# The Prognostic Value and Immune Infiltration of USP10 in Pan-Cancer: A Potential Therapeutic Target

**DOI:** 10.3389/fonc.2022.829705

**Published:** 2022-03-31

**Authors:** Dacheng Gao, Zhiwen Zhang, Rui Xu, Ziyang He, Fangyi Li, Yan Hu, Hui Chen, Jiawei Lu, Xingguo Cao, Yali Liu, Zengguang Xu

**Affiliations:** ^1^Shanghai East Hospital, Postgraduate Training Base of Jinzhou Medical University, Shanghai, China; ^2^Research Center for Translational Medicine, Shanghai East Hospital, School of Medicine, Tongji University, Shanghai, China; ^3^Shanghai East School of Clinical Medicine, Jinzhou Medical University, Shanghai, China

**Keywords:** USP10, prognosis, immune infiltration, pan-cancer, biomarker

## Abstract

Ubiquitin-specific peptidase 10 (USP10) can sustain cellular functions and regulate cellular processes. It plays an essential role in cancer inhibition or facilitation by reversing ubiquitin-proteasome degradation. Studies have identified USP10 to be involved in tumor progression in various cancers. However, the pan-cancer expression pattern of USP10, its prognostic value, and the association between tumor immune cell infiltration and USP10 expression remain to be discussed and thus comprised the aims of the present study. Based on clinical samples and bioinformatic analyses, high *USP10* expression was observed in most cancer tissues except for ovarian cancer. High *USP10* expression correlated with pathological stage and node metastasis and predicted poor patient prognosis. In addition, further analyses at the TIMER and GEPIA databases showed that USP10 is involved in the infiltration of multiple immune cells and regulated the infiltration levels of specific immune cell subpopulations, particularly in pancreatic adenocarcinoma (PAAD) and liver hepatocellular carcinoma (LIHC). Importantly, USP10 might influence survival by modulating immune infiltration in patients with PAAD and LIHC. These results identified USP10 as a potential biomarker for pan-cancer prognosis, and in certain cancers, USP10 could identify clinical prognosis linked to tumor immune infiltration.

## Introduction

Currently, in many human populations and regions, the leading cause of death is cancer, representing a severe threat to human health (1). The malignant phenotype of neoplasms usually correlates with dysregulation of protein synthesis (2). Proteostasis disorders and aberrant gene expression in cancer affect the patients’ clinical outcomes. In addition, there has been increasing interest in the role of the tumor microenvironment (TME) in cancer metastasis, in which the infiltration levels of dendritic cells, neutrophils, macrophages, T cells, and B cells, vary significantly. Targeting of immune cells that infiltrate the TME using immunotherapy has become a landmark in the history of tumor therapy and has dramatically advanced the development of oncological immunology (3).

There are various types of post-translational modifications of proteins. Among them, ubiquitination and deubiquitination, which add or remove ubiquitin from target proteins to promote protein degradation or stabilization, are essential to regulate cell cycle processes, cell signaling, the DNA damage response, and the nuclear factor kappa B (NF-κB) pathway (4, 5). Protein ubiquitination is the result of the covalent modification of substrate proteins by ubiquitin-activating enzyme E1, ubiquitin-binding enzyme E2, and ubiquitin-ligase E3.

Deubiquitinating enzymes (DUBs) can mediate and regulate the reversible deubiquitination of substrate proteins and are important factors in regulating the ubiquitin system. On the one hand, they are involved in the recycling of ubiquitin molecules, processing ubiquitin precursors, and editing of ubiquitin chains to regulate the function of conjugated proteins. On the other hand, they can influence proteostasis by removing ubiquitin from substrates and exert pro- or anti-cancer effects (4, 6). In addition, the function of DUBs in cells can be regulated by modifications such as phosphorylation, ubiquitination, and sumoylation, which in turn affect their catalytic activity, cellular localization, or protein abundance.

Ubiquitin-specific peptidase 10 (USP10) is a crucial DUB that is primarily localized in the cytoplasm. The *USP10* gene is situated on chromosome 16q24.1 and encodes a protein product comprising 798 amino acids (relative molecular mass = ~ 93 KDa) (7, 8). Its molecular structure is mainly that of a cysteine-type endopeptidase and a ubiquitin sulfhydryl esterase. USP10 acts as a regulator of the cell cycle and autophagy by deubiquitinating various proteins that are post-translationally transferred to the cytoplasm. USP10 specifically deubiquitinates and stabilizes P53. Under DNA damage stress conditions, USP10 is stabilized by Ataxia Telangiectasia mutated (ATM) kinase phosphorylation modification at Thr42 and Ser337, which drives its entry into the nucleus where it deubiquitinates P53, thereby regulating the P53 downstream network functions (9). In addition, by acting on wild-type P53, USP10 can exercise cancer suppressive functions; however, for some mutant P53s, USP10 might exert cancer-promoting functions.

Studies have shown that the abnormal expression of USP10 in different types of cancer correlates strongly with patient prognosis. High USP10 expression in prostate cancer, breast cancer, non-small cell lung cancer, colon cancer, and melanoma is associated with poor patient prognosis (10–14). However, low expression of USP10 predicts a poor prognosis in patients with ovary cancer (15). Furthermore, USP10 could deubiquitinate sirtuin 6 (SIRT6) to antagonize transcriptional activation of c-Myc oncogenes to inhibit tumor formation (16). In non-small cell lung cancer with mutant P53, targeting USP10 could boost drug sensitivity in patients with lung cancer (17). By contrast, USP10 is closely associated with tumor immunity. USP10 has been identified to be involved in metastasis and can drive tumor-associated macrophage polarization in colorectal cancer (18). Depletion of USP10 markedly reduced apoptosis and immune cell infiltration (19). It also stimulates the production of reactive oxygen species (ROS) in T cells, thereby promoting malignant mutations (20). These studies suggest that USP10 has a critical function in the initiation and progression cancer, and in tumor immunity. Therefore, the design of highly selective inhibitors might bring new hope for anti-cancer immunotherapy.

However, to date, USP10 has not been studied in pan-cancer. Its potential prognostic value and the relevance of immune infiltration are unclear, and there is a lack of macroscopic presentation and discussion. Therefore, this study aimed to determine whether USP10 influences the prognosis of patients with cancer and if such an influence is associated with immune cell infiltration.

## Materials and Methods

### Oncomine Database

The Oncomine cancer database was used to analyze *USP10* mRNA expression levels in different cancer types (https://www.oncomine.org/resource/login.html) (21). The threshold was set as a *P*-value of 0.001, a fold-change of 1.5, and the gene rank of ‘all’.

### TIMER Database

TIMER (https://cistrome.shinyapps.io/timer/) (22) enables the systematic analysis of immune cell infiltration in various types of cancer. Using TIMER, the association between *USP10* expression in different tumors and six types of immune infiltrates (including B cells, CD4+ T cells, CD8+ T cells, macrophages, neutrophils, and dendritic cells) was explored, as well as the correlation between *USP10* expression and immune cell gene markers after determining the tumor purity. Finally, we determined the associations between the expression of *USP10* and the genetic markers of specific subpopulations of immune infiltrating cells.

### HPA Database

The Human Protein Atlas (HPA) (http://www.proteinatlas.org/) was used to assess differences in USP10 expression at the protein level. This database contains immunohistochemical (IHC) data for USP10 protein levels in eight cancer tissues and their normal counterparts (breast, liver, lung, skin, colon, kidney, ovarian, and prostate cancers).

### GEPIA Database

Gene Expression Profiling Interactive Analysis (GEPIA) (http://gepia.cancer-pku.cn/) (23) is an online interactive web server for the analysis of tumor samples from The Cancer Genome Atlas (TCGA) and RNA sequencing expression data from the Genotype-Tissue Expression (GTEx) project. We used GEPIA to evaluate the association of *USP10* expression with prognosis in various tumor types, including overall survival (OS) and relapse-free survival (RFS). We further estimated the interaction between *USP10* expression and specific markers related to tumor immune cell infiltration.

### PrognoScan Database

The PrognoScan database (http://www.abren.net/PrognoScan/) (24) was used to assess the correlation between *USP10* expression and survival rate in different types of cancer, and to explore the prognostic value of USP10.

### Kaplan-Meier Plotter Database

The Kaplan-Meier plotter database (http://kmplot.com/analysis/) (25) uses meta-analysis data to discover and validate prognosis-related biomarkers. The association between *USP10* expression and patient survival in pan-cancer was investigated.

### cBioPortal Database

The open web resource cBioPortal database (http://www.cbioportal.org/) (26) is used to explore multidimensional cancer genome datasets. We used cBioPortal to analyze the impact of *USP10* mutations and copy number variation on various cancers.

### UALCAN Database

The UALCAN (http://ualcan.path.uab.edu/) (27) database contains valuable cancer OMICS data and information to help understand tumor staging and node metastasis related to the USP10 protein in a diverse range of cancers, which could inform mechanistic studies.

### Tissue Specimens

From May 2008 to Oct 2015, we collected tumors and adjacent non-tumor tissues from 12 patients suffering from different types of cancer who underwent surgical treatment in the Shanghai East Hospital Affiliated to Tongji University. The Ethics Committee of Shanghai East Hospital Affiliated to Tongji University approved this study (No. 2019tjdx110). Exemption from informed consent was granted because of the retrospective nature of the study.

### Hematoxylin and Eosin (H&E) Staining

Cancerous and control tissues from the breast, liver, lung, colorectum, kidney, ovary, prostate, stomach, skin, cerebrum, esophagus, and uterus were fixed in 4% paraformaldehyde. After deparaffinization and rehydration, sections were cuts at 4 μm thick and stained with H&E according to the manufacturer’s protocol (C0105S, Beyotime, Jiangsu, China). The sections were finally dehydrated and sealed for viewing under a light microscope (×200, Leica DM3000; Wetzlar, Germany).

### Immunofluorescence Staining

All tumor sections (4 μm) were deparaffinized and hydrated, quenched in 3% H_2_O_2_, immersed in citrate buffer, heated to retrieve the antigen, and then stained using immunofluorescence. USP10 primary antibodies (Invitrogen, Waltham, MA, USA; Cat. #PA5-52334) and fluorescently-conjugated secondary antibodies were applied, followed by 4′,6-diamidino-2-phenylindole (DAPI) staining to visualize the nuclei. The sections were observed under a microscope (×200, Leica DM3000).

### Statistical Analysis

Data in Oncomine are presented based on gene ranking, fold-change, and *P*-values. Survival curves were plotted using PrognoScan, Kaplan-Meier plotter, and GEPIA, and the results are displayed as the hazard ratio (HR) and *P*-value, or the *P*-value alone, from a log-rank test. Gene expression correlation was estimated using Spearman’s correlation, and r values were used to determine the magnitude of the correlation. *P*-values less than 0.05 were considered statistically significant.

## Results

### Transcriptional and Translational Levels of USP10 in Pan-Cancer

**Figure 1:**
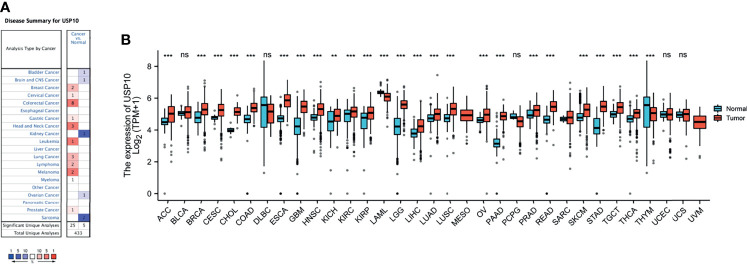
The mRNA expression level of *USP10* in pan-cancer. **(A)** Oncomine data showing enhanced or reduced *USP10* expression in various tumor tissue types and in normal tissues. **(B)** TGCA data showing the expression levels of human *USP10* in different types of cancer, with GTEx data as a control. **P < 0.01; ***P < 0.001. NS, no significance.

In the Oncomine database, the levels of *USP10* mRNA in various cancers and normal tissues were analyzed. The results showed that *USP10* expression in breast cancer, cervical cancer, colorectal cancer, gastric cancer, head and neck cancer, leukemia, lung cancer, lymphoma, melanoma, myeloma, and prostate cancer was higher than that in normal tissues (**Figure 1A**). By contrast, in some datasets, lower *USP10* expression was observed for brain and CNS cancer, bladder cancer, kidney cancer, ovarian cancer, and sarcoma. **Supplementary Table 1** shows the detailed results for the expression of *USP10* in various cancer types.

Next, TCGA RNA-seq data from multiple malignancies was analyzed for *USP10* expression (**Figure 1B**). Significantly higher *USP10* expression was detected in adrenocortical carcinoma (ACC), breast invasive carcinoma (BRCA), cervical squamous cell carcinoma and endocervical adenocarcinoma (CESC), cholangiocarcinoma (CHOL), colon adenocarcinoma (COAD), (esophageal carcinoma (ESCA), glioblastoma multiforme (GMB), (head and neck cancer (HNSC), (kidney chromophobe (KICH), kidney renal clear cell carcinoma (KIRC), kidney renal papillary cell carcinoma (KIRP), brain lower grade glioma (LGG), liver hepatocellular carcinoma (LIHC), lung adenocarcinoma (LUAD), lung squamous cell carcinoma (LUSC), ovarian serous cystadenocarcinoma (OV), pancreatic adenocarcinoma (PAAD), prostate adenocarcinoma (PRAD), rectum adenocarcinoma (READ), skin cutaneous melanoma (SKCM), stomach adenocarcinoma (STAD), testicular germ cell tumors (TGCT), and thyroid carcinoma (THCA) tissues compared with that in adjacent normal tissues. However, significantly lower *USP10* expression was observed in acute myeloid leukemia (LAML) and thymoma (THYM) compared with that in adjacent normal tissues. The analyses of the two databases were relatively consistent, except for KIRC and OV. These analytical differences were mainly due to the different databases and different sample sizes.

**Figure 2:**
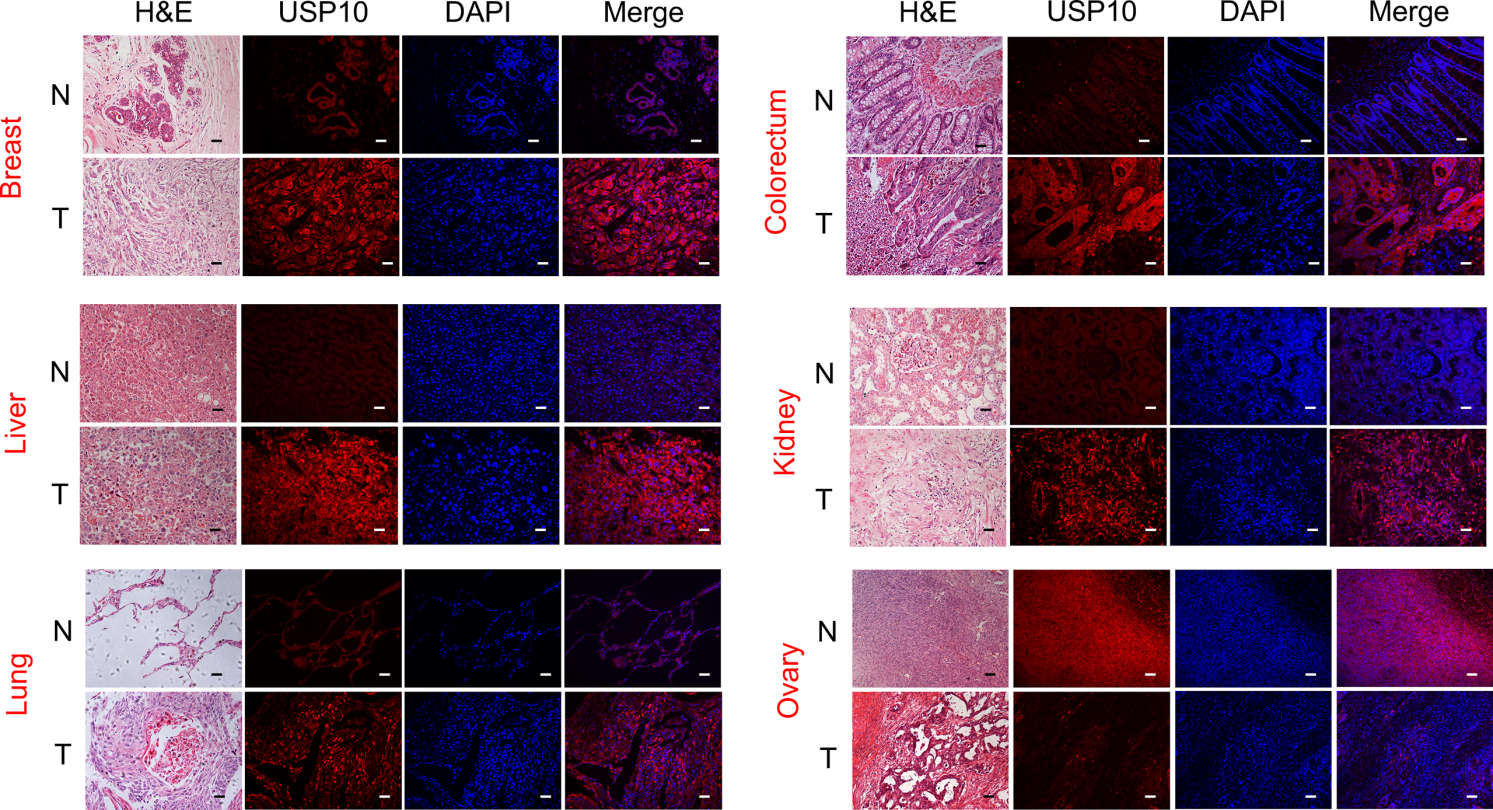
The protein level of USP10 in different types of cancer. Representative images of H&E stained normal and tumor slides (left 1st). Immunofluorescence staining analysis (left 2nd to 4th) showing that an evident fluorescent signal in the tumor tissue sections compared with that in the control group. However, in ovarian cancer, normal tissue has a stronger fluorescent signal than tumor tissue. All scale bars = 50 μm. H&E, hematoxylin-eosin staining. N, normal tissues; T, tumor tissues.

We used immunofluorescence (IF) to examine the expression of USP10 protein in various cancer tissues and their normal counterparts. USP10 protein expression was higher in breast cancer, liver cancer, lung cancer, colorectal cancer, kidney cancer, prostate cancer, stomach cancer, skin cancer, cerebrum cancer, esophagus cancer, and uterus cancer tissues than in normal tissues (**Figure 2** and **Supplementary Figure 1**). However, USP10 protein levels were lower in ovarian cancer tumor tissues compared with those in normal tissues (**Figure 2**), which was in line with the results for the mRNA levels from the Oncomine database (**Figure 1A**). Furthermore, the immunohistochemistry (IHC) results from the HPA database shown in **Supplementary Figure 2** showed similar results. Normal lung, prostate, and liver tissues showed moderate USP10 IHC staining, whereas tumor tissues showed intense staining. Normal breast, colon, kidney, and skin tissue samples showed weak USP10 staining, while tumor tissues showed intense staining. Interestingly, the normal ovary tissue sample had moderate USP10 staining, while tumor tissue had low staining (**Supplementary Figure 2**). These results suggested that USP10 protein levels are generally upregulated in the above tumor tissues but downregulated in ovarian cancer. The transcriptional and translational levels of USP10 in these cancer types were broadly consistent.

### Diagnostic Value of USP10 in Representative Tumors

**Figure 3:**
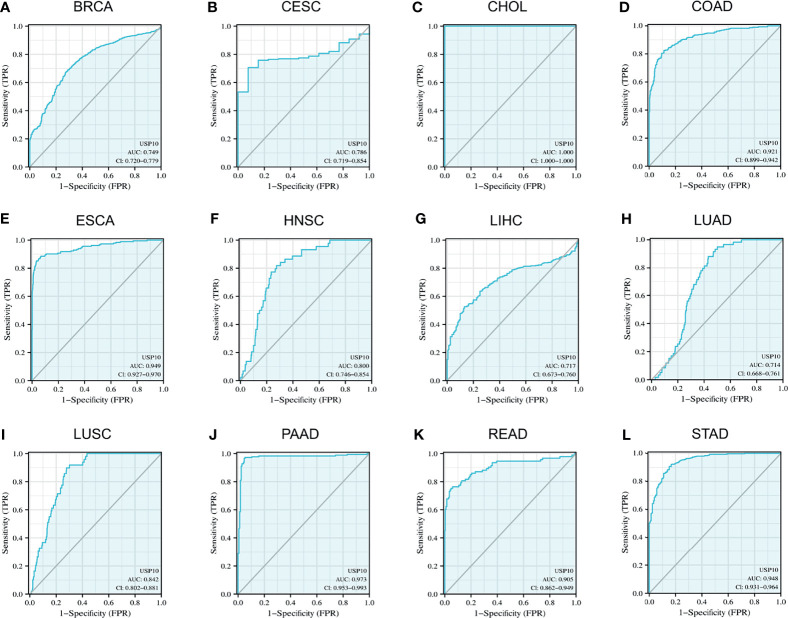
Receiver operating characteristic (ROC) curves for *USP10* in representative tumors. **(A)** BRCA, **(B)** CESC, **(C)** CHOL, **(D)** COAD, **(E)** ESCA, **(F)** HNSC, **(G)** LIHC, **(H)** LUAD, **(I)** LUSC, **(J)** PAAD, **(K)** READ, **(L)** STAD. Values under the ROC curve ranges from 0.5 to 1. The closer the area under the curve (AUC) is to 1, the better the diagnosis. The horizontal coordinate is the False Positive Rate (FPR), and the vertical coordinate is the True Positive Rate (TPR).

Given that USP10 expression was upregulated in a variety of cancers, we used ROC curves to assess the diagnostic value of USP10 for pan-cancer. The results revealed that USP10 had a certain accuracy (area under the ROC curve (AUC) > 0.7) to predict 12 representative tumors, including BRCA (AUC = 0.749) (**Figure 3A**), CESC (AUC = 0.786) (**Figure 3B**), CHOL (AUC = 1.000) (**Figure 3C**), COAD (AUC = 0.921) (**Figure 3D**), ESCA (AUC = 0.949) (**Figure 3E**), HNSC (AUC = 0.800) (**Figure 3F**), LIHC (AUC = 0.717) (**Figure 3G**), LUAD (AUC = 0.714) (**Figure 3H**), LUSC (AUC = 0.842) (**Figure 3I**), PAAD (AUC = 0.973) (**Figure 3J**), READ (AUC = 0.905) (**Figure 3K**), and STAD (AUC = 0.948) (**Figure 3L**). Among them, USP10 had high accuracy (AUC > 0.9) for PAAD, CHOL, ESCA, STAD, and READ. These results suggested that USP10 has a different diagnostic value depending on the type of cancer.

### Prognostic Value of USP10 in Different Cancers

**Figure 4:**
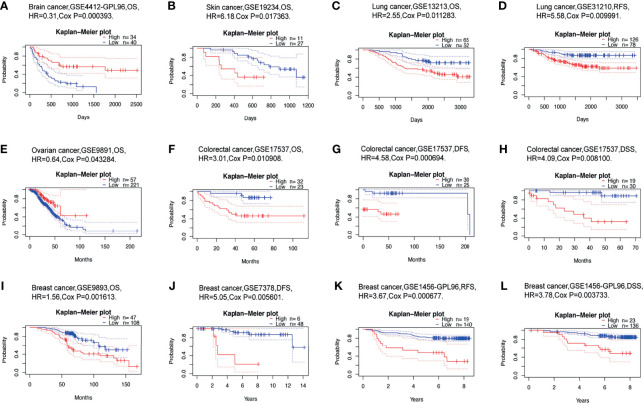
Correlation between *USP10* expression and the prognosis of various types of cancer using data from the PrognoScan database. **(A)** Overall survival (OS) of a cohort of 74 patients with brain cancer (GSE4412-GPL96). **(B)** OS of a cohort of 38 patients with skin cancer (GSE19234). **(C, D)** OS and relapse-free survival (RFS) survival curves for patients in two lung cancer cohorts [GSE13213 (n=117) and GSE31210 (n=204)]. **(E)** OS of a cohort of 278 patients with ovarian cancer (GSE9891). **(F–H)** OS (n = 55), disease-free survival (DFS) (n = 55), disease free survival (DSS) (n = 49) survival curves for a cohort of patients with colorectal cancer (GSE17537). **(I, J)** OS and DFS survival curves for two cohorts of patients with breast cancer [GSE9893 (n =155) and GSE7378 (n =54)]. **(K, L)** RFS (n =159) and DSS (n =159) survival curves for a cohort of patients with breast cancer (GSE1456-GPL96). Patients with high *USP10* expression are represented by the red curve. Significance is indicated by a *P*-value < 0.05.

We than investigated whether USP10 expression was linked to the prognosis of patients with cancer. The impact of *USP10* expression on survival rates was evaluated using PrognoScan, primarily using Gene Expression Omnibus (GEO) data. Notably, the expression of *USP10* had a marked effect on the prognosis of six types of cancers, including brain cancer, skin cancer, lung cancer, ovarian cancer, colorectal cancer, and breast cancer (**Figures 4A–L**). In patients with these cancers, high *USP10* expression could be an independent risk factor. Details of the association between *USP10* expression and the prognosis of different cancers are shown in **Supplementary Table 2**.

**Figure 5:**
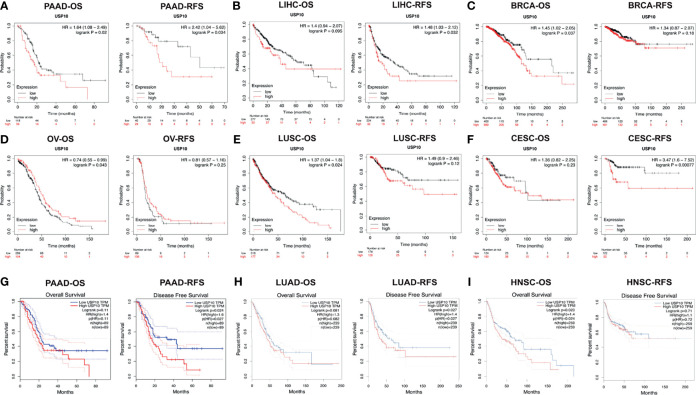
Correlation between *USP10* expression and the prognosis of various types of cancer in the Kaplan–Meier Plotter **(A–F)** and GEPIA **(G–I)** databases. Overall survival (OS) and relapse-free survival (RFS) survival curves of patients with **(A)** pancreatic cancer (PAAD), **(B)** liver cancer (LIHC), **(C)** breast cancer (BRCA), **(D)** ovarian cancer (OV), **(E)** lung squamous cell carcinoma (LUSC), **(F)** cervical squamous cell carcinoma and endocervical adenocarcinoma (CESC), **(G)** pancreatic cancer (PAAD), **(H)** lung adenocarcinoma (LUAD), **(I)** head and neck squamous cell carcinoma (HNSC). Patients with high *USP10* expression are represented by the red curve. Significance is indicated by a *P*-value < 0.05.

To further investigate USP10’s prognostic potential various cancers, Kaplan-Meier Plotter was employed, which mainly uses TCGA data from Affymetrix microarrays. **Figures 5A–F** show the details of the expression of *USP10* in the different types of cancer. High *USP10* expression correlated with unfavorable prognosis of in terms of OS and RFS in PAAD; OS in BRCA and LUSC; and RFS in LIHC and CESC. However, a favorable prognosis in terms of OS was related to high *USP10* expression in OV. The analysis revealed that USP10 mRNA and protein levels were downregulated significantly in ovarian tumor tissues compared with those in normal tissues, but high USP10 expression improved the OS of patients with ovarian cancer significantly (**Figures 4E**, **5D**). In addition, we examined USP10-related survival (OS and RFS) using the GEPIA database (**Figures 5G–I**). Poor prognosis in PAAD (RFS, HR = 1.6, *P* = 0.027), LUAD (RFS, HR = 1.4, *P* = 0.027), and HNSC (OS, HR = 1.4, *P* = 0.024) correlated with higher *USP10* expression. These results suggest that *USP10* expression has different prognostic values depending on the cancer type.

### Genetic Alterations of *USP10* in Pan-Cancer and TMB-Based Survival Analysis of *USP10* Expression in Patients With Tumors

**Figure 6:**
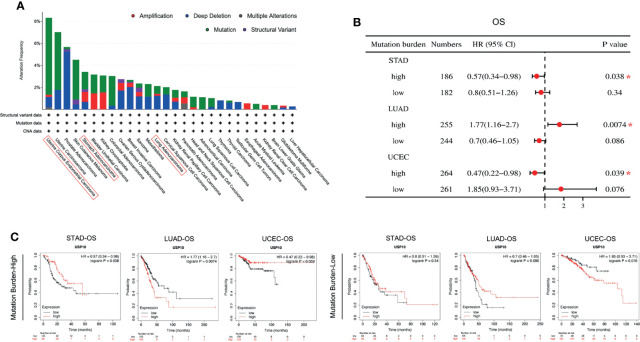
cBioPortal access of TGCA data showing *USP10* mutation features in pan-cancer correlation of the TMB and *USP10* in Kaplan–Meier Plotter. **(A)** Mutation type alteration frequencies. **(B)** The potential associations between the TMB and *USP10* expression in the OS of patients with cancer, shown as a forest plot. **(C)** Survival curves between the TMB and USP10 expression in the OS of patients with cancer. TMB, tumor mutation burden. STAD, stomach adenocarcinoma; LUAD, lung adenocarcinoma; UCEC, uterine corpus endometrial carcinoma. OS, overall survival. *P < 0.05.

Tumorigenesis is usually accompanied by genetic alterations. Therefore, the genetic alterations of *USP10* were examined in various tumor samples in the TCGA database (**Figure 6A**). *USP10* showed the highest alteration frequency (6.99%) in patients with endometrial tumors with “mutation” as the primary type. Copy number alterations (CAN) of the “deep deletion” type were the predominant type of mutation in prostate adenocarcinoma cases, with a frequency of 5.26%. Noticeably, almost all the tumor cases with genetic alterations had deletions or mutations of *USP10* (**Figure 6A**). *USP10* gene mutations were observed in numerous cancers; therefore, we next explored the potential relationship between the tumor mutational burden (TMB) and *USP10* expression in the clinical survival prognosis of different types of cancer (**Figure 6B**). *USP10* expression correlated significantly with the OS of patients with STAD (HR = 0.57, *P* = 0.038) and UCEC (HR = 0.47, *P* = 0.039) with a high TMB, showing a better prognosis compared with subjects with a low TMB (**Figure 6C**). By contrast, the expression of *USP10* correlated significantly with OS in patients with LUAD (HR = 1.77, *P* = 0.0074) with a high TMB; however, their prognosis was worse compared with patients with a low TMB (**Figure 6C**). These results suggested that patient prognosis is affected by the association between *USP10* expression and the TMB in certain cancers. Moreover, genetic alterations in *USP10* might play an important role in the genomes of endometrial and prostate cancers. These findings warrant further in-depth investigation.

### *USP10* Expression and Clinical Parameters of Patients With Different Cancers

**Figure 7:**
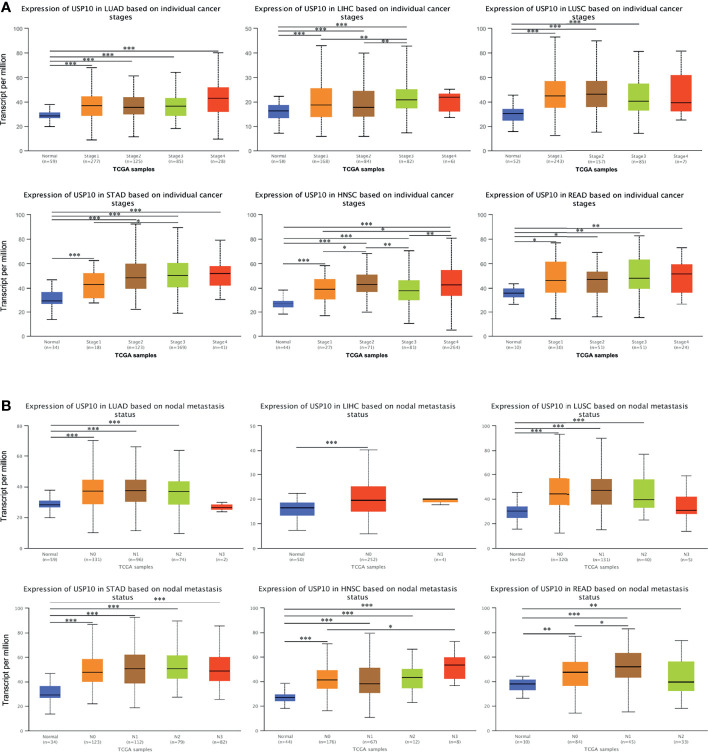
UALCAN database analysis of correlation between clinicopathological parameters and *USP10* expression in multiple cancer types. **(A)** Association between *USP10* expression and tumor stages in patients with different types of cancer. **(B)** The association between *USP10* expression and nodal metastasis status in patients with different types of cancer. LUAD, lung adenocarcinoma; LIHC, liver hepatocellular carcinoma; LUSC, lung squamous cell carcinoma; STAD, stomach adenocarcinoma; HNSC, head and neck squamous cell carcinoma; READ, rectal adenocarcinoma. *P < 0.05; **P < 0.01; ***P < 0.001.

To obtain a more detailed understanding of the role of USP10 in the progression of cancers, *USP10* expression was analyzed in patients with multiple cancers based on different clinical parameters using UALCAN. In terms of tumor staging, the expression of *USP10* was increased significantly in patients with LUSC at stages 1, 2, and 3; and in patients with READ and LUAD at stages 1, 2, 3, and 4 (**Figure 7A**). Moreover, patients with STAD had markedly elevated *USP10* expression in stages 1, 2, 3, and 4; and the changes at stage 1 and 3 were statistically significance (*P* < 0.05) (**Figure 7A**). The expression of *USP10* was upregulated in patients with LIHC. Interestingly, with the deterioration of LIHC, *USP10* expression increased gradually (**Figure 7A**). Notably, patients with HNSC showed significant overexpression of *USP10* in stages 1, 2, 3, and 4. In addition, the comparisons of stage 1 *vs.* 2, stage 1 *vs.* 4, stage 2 *vs.* 3, and stage 3 *vs.* 4 were also statistically significant. However, *USP10* was downregulated significantly in stage 3, possibly influenced by different molecular signaling pathways or different molecular subtypes (**Figure 7A**). For lymph node metastasis, patients with LUSC, READ, and LUAD with N0, N1, or N2 metastasis, *USP10* expression was higher than that in patients with other stages of metastasis (**Figure 7B**). The same *USP10* expression pattern was observed for patients with STAD and HNSC N0, N1, N2, or N3 metastasis and in patients with LIHC with N0 metastasis (**Figure 7B**). Thus, the expression of *USP10* correlated closely with the proliferation and lymph node metastasis of tumors.

### The Association of *USP10* Expression With Immune Cell Infiltration in Various Cancers

Numerous inflammatory and immune cells infiltrate cancer tissue, and recent studies have revealed the importance of tumor-infiltrating lymphocytes (TILs) in predicting the prognosis of patient survival (28–31). Therefore, the relationship between *USP10* expression and immune cell infiltration in 39 types of cancer was assessed using the TIMER database. (**Supplementary Figure 3**). Notably, in 14 cancers, the expression of *USP10* correlated significantly with B cell infiltration levels. Moreover, the expression of *USP10* correlated strongly with the infiltration level of dendritic cells in 10 cancers, with neutrophils in 24 cancers, with macrophages in 18 cancers, with CD4+ T cells in 13 cancers, and with CD8+ T cells in 15 cancers. Notably, *USP10* expression levels in ACC, CHOL, HNSC-HPV+, UCEC, and UCS were not significantly associated with the infiltration of B cells, CD4+ T cells, CD8+ T cells, macrophages, neutrophils, and dendritic cells (**Supplementary Figure 3**). Thus, USP10 might exert a fundamental function in the TME of cancers.

**Figure 8:**
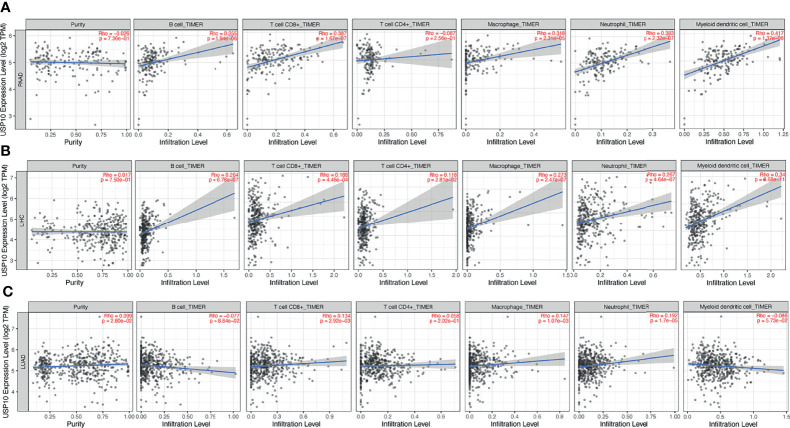
TIMER database analysis of the correlation between *USP10* expression and immune infiltration level in LUAD, LIHC, and PAAD. **(A)** In PAAD, *USP10* expression was not related to tumor purity, but correlated significantly and positively with infiltration levels of dendritic cells, neutrophils, macrophages, CD8+ T cells, and B cells. **(B)** In LIHC, *USP10* expression was not related to tumor purity, but correlated significantly and positively with dendritic cells, neutrophils, macrophages, CD4+ T cells, CD8+ T cells, and B cells. **(C)** In LUAD, *USP10* expression correlated weakly with tumor purity and correlated significantly and positively with CD8+ T cells, macrophages, and neutrophils, but had no significant relationship with the immune infiltration of B cells, CD4+ T cells, and dendritic cells. PAAD, pancreatic adenocarcinoma; LIHC, liver hepatocellular carcinoma; LUAD, lung adenocarcinoma. Significance is indicated by a *P*-value < 0.05.

The correlation between the expression of *USP10* and immune cell infiltration in various cancers prompted us to identify those cancers in which prognosis and immune infiltration were associated with *USP10* expression. The effect of immunotherapy and immune cell infiltration can be better evaluated in cancers for which the tumor purity has been determined (32, 33). Therefore, we selected PAAD, LIHC, and LUAD cancers for validation. After determining the tumor purity, we found that for PAAD, high expression of *USP10* correlated significantly and positively with the infiltration levels of CD8+ T cells (r = 0.387, *P* = 1.67e-07), B cells (r = 0.355, *P* = 1.94e-06), macrophages (r = 0.318, *P* = 2.31e-05), neutrophils (r = 0.383, *P* = 2.32e-07), and dendritic cells (r = 0.417, *P* = 1.37e-08) (**Figure 8A**). Similarly, there were significant positive correlations with the infiltration levels of dendritic cells (r = 0.34, *P* = 8.68e-11), neutrophils (r = 0.267, *P* = 4.64e-07), macrophages (r = 0.273, *P* = 2.47e-07), CD4+ T cells (r = 0.118, *P* = 2.81e-02), CD8+ T cells (r = 0.188, *P* = 4.46e-04), and B cells (r = 0.264, *P* = 6.78e-07), in LIHC (**Figure 8B**). However, *USP10* expression correlated weakly with immune cell infiltration in LUAD, including only CD8+ T cells (r = 0.134, *P* = 2.92e-03), macrophages (r = 0.147, *P* = 1.07e-03), and neutrophils (r = 0.192, *P* = 1.7e-05) (**Figure 8C**). These findings suggested that USP10 might play an important part in immune cell infiltration in these cancers.

### Assessment of Correlations Between Immune Cell Markers and *USP10* Expression

Next, we used the TIMER database to investigate potential correlations between USP10 and immune cell marker genes in PAAD, LIHC, and LUAD, such as CD8+ T cells, B cells, tumor-associated macrophages (TAMs), monocytes, M1/M2 macrophages, natural killer (NK) cells, DCs, neutrophils, general T cells, and T cell with different functions, e.g., T follicular helper (Tfh) cells, T helper type 1 (Th1) cells, T helper type 2 (Th2) cells, T helper type 17 (Th17) cells, regulatory T cells (Tregs), and exhausted T cells (**Table 1**). The results, which were adjusted for tumor purity, showed that in PAAD and LIHC, the expression level of *USP10* correlated significantly with most immune markers for the various immune cells. However, in LUAD, the expression level of *USP10* was only associated with 23 marker genes (**Table 1**).

**Table 1:** Analysis of correlations between *USP10* expression and immune cell markers by TIMER.

Description	Gene markers	PAAD				LIHC				LUAD			
		None		Purity		None		Purity		None		Purity		
		Cor	*P*	Cor	*P*	Cor	*P*	Cor	*P*	Cor	*P*	Cor	*P*	
**CD8+ T cells**	CD8A	0.214	*	0.189	0.013	0.173	**	0.191	**	0.071	0.106	0.13	*	
	CD8B	0.251	**	0.22	*	0.038	0.388	0.107	0.047	0.038	0.388	0.073	0.107	
**T cells (general)**	CD3D	0.154	0.039	0.12	0.118	0.067	0.196	0.082	0.127	-0.082	0.062	-0.031	0.494	
	CD3E	0.194	*	0.162	0.035	0.129	0.013	0.156	*	-0.005	0.910	0.063	0.161	
	CD2	0.202	*	0.174	0.023	0.1	0.055	0.121	0.024	-0.028	0.520	0.038	0.398	
**B cells**	CD19	0.146	0.051	0.115	0.134	0.143	*	0.128	0.018	-0.036	0.412	0.002	0.968	
	CD79A	0.157	0.035	0.118	0.123	0.077	0.136	0.085	0.114	-0.052	0.236	-0.019	0.681	
**Monocytes**	CD86	0.289	***	0.267	**	0.279	***	0.329	***	-0.055	0.213	0	0.991	
	CD115 (CSF1R)	0.257	**	0.255	**	0.266	***	0.315	***	-0.003	0.938	0.051	0.255	
**TAMs**	CCL2	0.016	0.832	-0.012	0.873	0.184	**	0.223	***	-0.02	0.656	0.019	0.675	
	CD68	0.325	***	0.312	***	0.225	***	0.245	***	-0.015	0.741	0.037	0.416	
	IL10	0.201	*	0.173	0.024	0.245	***	0.27	***	-0.037	0.408	0.014	0.765	
**M1 Macrophages**	INOS (NOS2)	0.224	*	0.184	0.016	0.149	*	0.158	*	0.085	0.055	0.098	0.030	
	IRF5	0.261	**	0.237	*	0.33	***	0.323	***	-0.042	0.346	-0.007	0.872	
	COX2 (PTGS2)	0.337	***	0.33	***	0.285	***	0.353	***	0.089	0.044	0.081	0.072	
**M2 Macrophages**	CD163	0.342	***	0.337	***	0.277	***	0.317	***	0.092	0.037	0.158	**	
	VSIG4	0.26	**	0.261	**	0.218	***	0.259	***	-0.097	0.028	-0.053	0.239	
	MS4A4A	0.255	**	0.239	*	0.24	***	0.284	***	-0.08	0.068	-0.026	0.564	
**Neutrophils**	CD66b (CEACAM8)	0.149	0.046	0.124	0.106	0.034	0.514	0.046	0.398	-0.063	0.153	-0.052	0.251	
	CD11b (ITGAM)	0.264	**	0.244	*	0.361	***	0.397	***	-0.053	0.230	-0.001	0.984	
	CCR7	0.157	0.036	0.128	0.096	0.143	*	0.161	*	-0.012	0.785	0.047	0.295	
**Natural killer cells**	KIR2DL1	0.021	0.779	0.023	0.763	0.091	0.080	0.073	0.177	0.031	0.486	0.047	0.298	
	KIR2DL3	0.064	0.398	0.03	0.693	0.176	**	0.189	**	0.122	*	0.162	**	
	KIR2DL4	0.215	*	0.115	0.019	0.189	**	0.198	**	0.111	0.012	0.141	*	
	KIR3DL1	0.029	0.699	0.006	0.938	0.174	**	0.197	**	0.069	0.120	0.093	0.040	
	KIR3DL2	0.226	*	0.198	*	0.063	0.230	0.077	0.154	0.121	*	0.166	**	
	KIR3DL3	0.188	0.012	0.166	0.030	0.04	0.437	-0.016	0.774	0.081	0.067	0.095	0.035	
	KIR2DS4	0.105	0.163	0.078	0.313	0.101	0.052	0.114	0.034	0.056	0.208	0.087	0.053	
**Dendritic cells**	HLA-DPB1	0.159	0.034	0.126	0.101	0.211	***	0.238	***	-0.198	***	-0.17	**	
	HLA-DQB1	0.212	*	0.192	0.012	0.126	0.015	0.147	*	-0.197	***	-0.167	**	
	HLA-DRA	0.265	**	0.241	*	0.287	***	0.328	***	-0.205	***	-0.173	**	
	HLA-DPA1	0.259	**	0.234	*	0.274	***	0.312	***	-0.139	*	-0.109	0.015	
	BDCA-1 (CD1C)	0.137	0.068	0.116	0.132	0.124	0.017	0.142	*	-0.234	***	-0.208	***	
	BDCA-4 (NRP1)	0.362	***	0.388	***	0.541	***	0.552	***	0.142	*	0.164	**	
	CD11c (ITGAX)	0.15	0.045	0.104	0.177	0.312	***	0.355	***	-0.083	0.065	0.034	0.454	
**Th1 cells**	T-bet (TBX21)	0.108	0.149	0.091	0.237	0.139	*	0.161	*	0.077	0.082	0.152	**	
	STAT4	0.097	0.196	0.117	0.128	0.06	0.245	0.07	0.195	-0.073	0.096	-0.026	0.571	
	STAT1	0.412	***	0.396	***	0.385	***	0.392	***	0.263	***	0.322	***	
	IFN-γ (IFNG)	0.091	0.227	0.078	0.308	0.139	*	0.148	*	0.076	0.084	0.133	*	
	TNF-α (TNF)	0.083	0.269	0.069	0.372	0.26	***	0.289	***	-0.001	0.987	0.058	0.200	
**Th2 cells**	GATA3	0.213	*	0.2	*	0.221	***	0.267	***	0.088	0.046	0.15	**	
	STAT6	0.436	***	0.417	***	0.429	***	0.404	***	0.137	*	0.147	*	
	STAT5A	0.372	***	0.344	***	0.316	***	0.315	***	0.081	0.066	0.146	*	
	IL13	-0.019	0.799	-0.025	0.749	0.039	0.451	0.016	0.771	-0.073	0.097	-0.035	0.440	
**Tfh cells**	BCL6	0.458	***	0.444	***	0.432	***	0.427	***	0.155	**	0.156	**	
	IL21	0.183	0.015	0.161	0.036	0.087	0.096	0.103	0.057	0.138	*	0.168	**	
**Th17 cells**	STAT3	0.528	***	0.524	***	0.476	***	0.483	***	0.33	***	0.324	***	
	IL17A	0.1	0.182	0.1	0.194	0.086	0.097	0.096	0.075	0.001	0.981	0.018	0.686	
**Tregs**	FOXP3	0.235	*	0.213	*	0.257	***	0.278	***	0.019	0.671	0.08	0.075	
	CCR8	0.348	***	0.324	***	0.409	***	0.438	***	0.09	0.041	0.153	**	
	STAT5B	0.39	***	0.427	***	0.523	***	0.512	***	0.321	***	0.331	***	
	TGFβ (TGFB1)	0.129	0.086	0.095	0.216	0.294	***	0.334	***	0.01	0.818	0.048	0.287	
**Exhausted T cells**	PD-1 (PDCD1)	0.18	0.016	0.144	0.060	0.129	0.013	0.13	0.016	0.047	0.286	0.103	0.022	
	CTLA4	0.178	0.017	0.145	0.058	0.136	*	0.154	*	-0.009	0.845	0.063	0.163	
	LAG3	0.122	0.102	0.114	0.138	0.079	0.128	0.086	0.109	0.108	0.015	0.156	**	
	TIM-3 (HAVCR2)	0.247	**	0.226	*	0.287	***	0.342	***	-0.093	0.034	-0.04	0.379	
	GZMB	0.196	*	0.156	0.042	0.084	0.105	0.078	0.146	0.089	0.042	0.147	*	

PAAD, pancreatic adenocarcinoma; LIHC, liver hepatocellular carcinoma; LUAD, lung adenocarcinoma. TAM, tumor-associated macrophage; Treg, regulatory T cell; Tfh, follicular helper T cell; Th, T helper cell. Purity, adjusted correlation according to tumor purity; None, non-adjusted correlation. Cor, R value of Spearman’s correlation. *P < 0.01; **P < 0.001; ***P < 0.0001.

**Figure 9:**
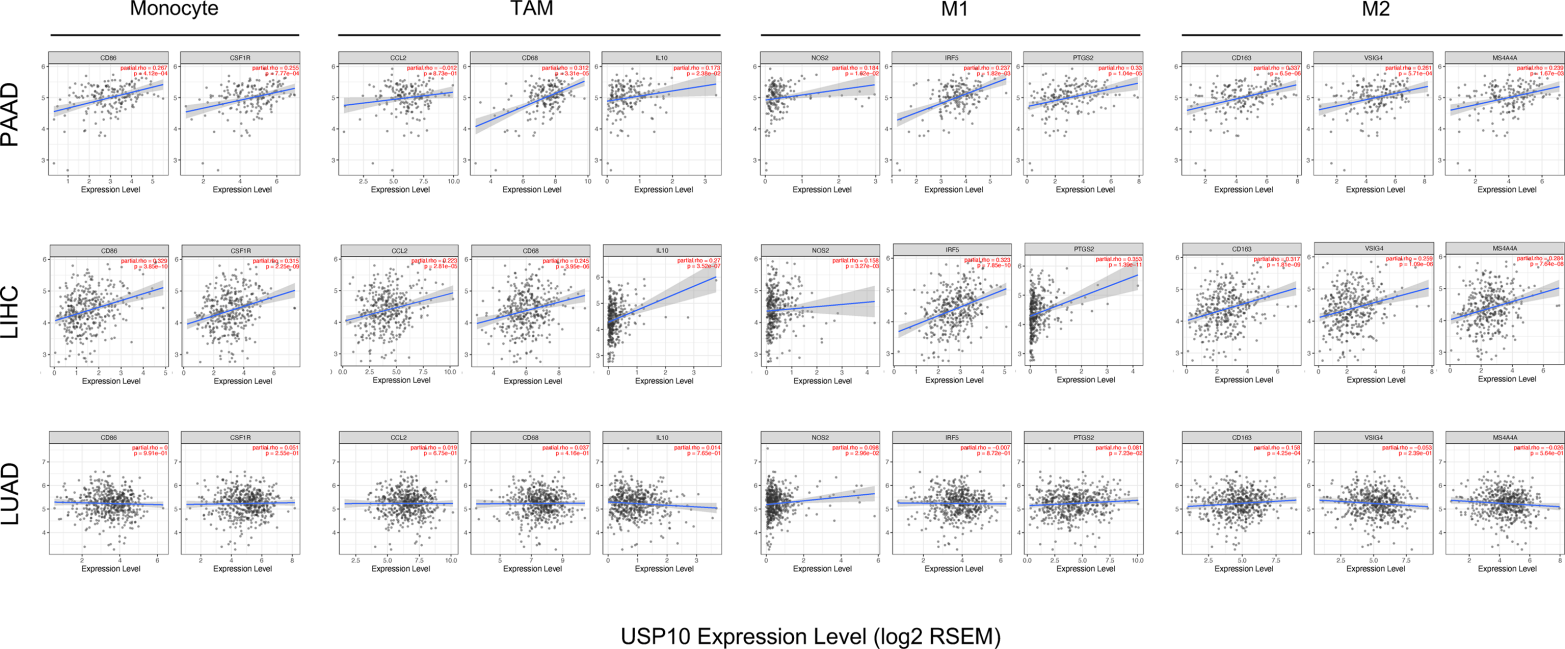
The correlation between the expression of *USP10* and marker genes for M2 macrophages, M1 macrophages, TAMs, and monocytes in LUAD, LIHC, and PAAD, as shown by scatter plots. The monocyte markers were *CSF1R* and *CD86*; the markers for TAMs were *CCL2*, *CD68*, and *IL10*; the markers for M1 macrophages were *PTGS2*, *IRF5*, and *NOS2*; and the markers for M2 macrophages were *MS4A4A*, *VSIG4*, and *CD163*. PAAD, pancreatic adenocarcinoma; LIHC, liver hepatocellular carcinoma; LUAD, lung adenocarcinoma. TAMs, tumor-associated macrophages. Significance is indicated by a *P*-value < 0.05.

Interestingly, USP10 expression correlated significantly with markers of Tregs (*CCR8* (encoding C-C motif chemokine receptor 8) and *STAT5B* (encoding signal transducer and activator of transcription 5B)) in LUAD, LIHC, and PAAD **(** **Table 1**). The expression of *USP10* was statistically significant for TAMs, monocytes, and M1/M2 macrophages in PAAD and LIHC, but not in LUAD (**Table 1**). Specifically, it was markedly correlated with marker genes of monocytes (*CD86*, *CSF1R* (encoding colony stimulating factor 1 receptor)), marker genes of TAMs (*CD68*, *IL10* (encoding interleukin-10)), marker genes of M1 macrophages (*NOS2* (encoding nitrous oxide synthase 2), *IRF5* (encoding interferon regulatory factor 5), and *PTGS2* (encoding prostaglandin-endoperoxide synthase 2)), marker genes of M2 macrophages (*CD163*, *VSIG4* (encoding V-set and immunoglobulin domain containing 4), and *MS4A4A* (encoding membrane spanning 4-domains A4A)) in PAAD and LIHC (**Figure 9**). Given the homologous data in GEPIA and TIMER from the TCGA, we used the GEPIA database to further assess the associations between the expression of *USP10* and markers genes of monocytes and TAMs in tumor tissues of PAAD, LIHC, and LUAD. Similar results to those obtained using TIMER were observed (**Table 2**). These results indicated that USP10 might participate in immune cell infiltration and regulate the polarization of macrophages in both PAAD and LIHC TMEs. The precise mechanism requires confirmation in further studies.

**Table 2:** Analysis of the correlations between *USP10* expression and genetic markers of monocytes and macrophages using GEPIA data.

Description	Gene markers	PAAD				LIHC				LUAD			
		Tumor		Normal		Tumor		Normal		Tumor		Normal		
		R	*P*	R	*P*	R	*P*	R	*P*	R	*P*	R	*P*	
**Monocytes**	CD86	0.340	***	-0.700	0.300	0.320	***	0.380	*	-0.033	0.470	-0.130	0.35	
	CD115 (CSF1R)	0.320	***	0.230	0.770	0.280	***	0.410	*	0.034	0.450	0.220	0.091	
**TAMs**	CCL2	0.130	0.079	-0.180	0.820	0.210	***	0.170	0.220	0.005	0.910	0.097	0.46	
	CD68	0.400	***	-0.940	0.058	0.230	***	0.430	*	0.064	0.160	0.017	0.9	
	IL10	0.180	0.015	-0.910	0.093	0.260	***	0.130	0.350	-0.042	0.360	-0.074	0.58	
**M1 Macrophages**	INOS (NOS2)	0.130	0.080	0.360	*	0.015	0.770	0.110	0.460	0.068	0.140	0.290	0.026	
	IRF5	0.320	***	-0.470	**	0.320	***	0.240	0.088	0.009	0.840	0.058	0.66	
	COX2 (PTGS2)	0.110	0.140	-0.850	0.150	0.170	*	0.120	0.400	0.097	0.034	0.350	*	
**M2 Macrophages**	CD163	0.310	***	0.037	0.960	0.190	**	0.330	0.018	0.027	0.550	-0.029	0.83	
	VSIG4	0.290	***	-0.980	0.017	0.210	***	0.350	0.013	-0.066	0.150	-0.200	0.13	
	MS4A4A	0.280	**	-0.950	0.055	0.220	***	0.370	*	-0.054	0.240	-0.200	0.14	

PAAD, pancreatic adenocarcinoma; LIHC, liver hepatocellular carcinoma; LUAD, lung adenocarcinoma. TAM, tumor-associated macrophage. Tumor, association analysis in tumor tissues from TCGA; Normal, association analysis in normal tissues from TCGA. *P < 0.01; **P < 0.001; ***P < 0.0001.

### Analysis of Survival Related to *USP10* Expression Based on Immune Cell Infiltration

**Figure 10:**
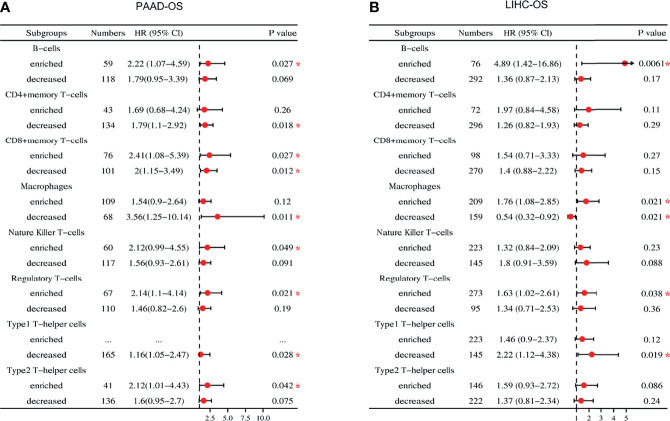
Forest plot of the prognostic value of the expression of *USP10* in immune cell subpopulations of patients with PAAD and LIHC. **(A)** In patients with PAAD, the prognostic benefit of *USP10* expression depends on the patients’ different immune cell subsets, according to the forest plot. **(B)** In patients with LIHC, there was an association between the expression of *USP10* and OS for the infiltration of different immune cell subgroups, according to a forest plot. PAAD, pancreatic adenocarcinoma; LIHC, liver hepatocellular carcinoma. OS, overall survival. *P < 0.05.

*USP10* expression correlated significantly with poor prognosis and immune infiltration of patients with PAAD and LIHC; therefore, we investigated whether *USP10* expression could affect the prognosis of patients with PAAD and LIHC *via* immune infiltration (**Figure 10**). Based on *USP10* expression in relevant immune cell subsets, we found that in PAAD, high *USP10* expression was linked to increased infiltration of B cells (OS, HR = 2.22, *P* = 0.027), NK cells (OS, HR = 2.12, *P* = 0.049), Tregs (OS, HR = 2.14, *P* = 0.021), and Th2 cells (OS, HR = 2.12, *P* = 0.042); and with decreased infiltration of CD4+ T cells, macrophages, and Th1 cells (all *P* < 0.05), and predicted inferior prognostic survival in patients with PAAD (**Figure 10A** and **Supplementary Figure 4A**). In LIHC, overexpression of *USP10* and abundant infiltration of B cells (OS, HR = 4.89, *P* = 0.0061) and Treg cells (OS, HR = 1.63, *P* = 0.038) or reduced infiltration of Th1 cells (OS, HR = 2.22, *P* = 0.019) predicted a worse prognosis (**Figure 10B** and **Supplementary Figure 4B**).

Interestingly, *USP10* expression with increased (OS, HR = 2.41, *P* = 0.027) or decreased (OS, HR = 2, *P* = 0.012) CD8+ T cell infiltration had a marked impact on the survival of patients with PAAD and predicted a worse survival outcome (**Figure 10A** and **Supplementary Figure 4A**). In addition, *USP10* expression in LIHC correlated statistically with enriched infiltration of macrophages (OS, HR = 1.76, *P* = 0.021), indicating a worse prognosis. In contrast, *USP10* expression was associated with reduced infiltration of macrophages (OS, HR = 0.54, *P* = 0.021), predicting a better survival outcome (**Figure 10B** and **Supplementary Figure 4B**). These results further demonstrated that USP10 might regulate macrophage polarization in the TME of LIHC and have an important impact on prognosis. Critically, the potential explanation for how USP10 influences the prognosis of patients with PAAD and LIHC might stem in part from immune infiltration.

## Discussion

USP10 specifically cleaves ubiquitin from ubiquitin-conjugated protein substrates and thus affects cellular processes. Although the aberrant expression of USP10 has been reported in many cancers (10–12, 14, 34, 35), and the role of USP10 in tumorigenesis and prognosis has been partially confirmed in several cancers (10–14), a systematic bioinformatic analysis is still lacking. Based on bioinformatics, this study investigated USP10 expression in pan-cancer and its correlation with prognosis and analyzed the importance of USP10 in the development of different cancers. Moreover, the association between *USP10* gene expression in the TME and immune cell infiltration was determined. Our findings provide useful insights to further explore the role of USP10 in tumorigenesis and progression *via* mechanistic studies.

In this study, we analyzed the differential expression of *USP10* and its prognostic value in different types of cancer. The results showed that *USP10* mRNA was highly expressed in most tumor types, except for bladder cancer (BLCA), brain and central nervous system cancer, OV, sarcoma (SARC), acute myeloid leukemia (AML), and THYM. The results of the current study suggest that USP10 acts as a therapeutic target for PRAD and AML (36, 37). In addition, IF analysis of 12 clinical samples and the IHC images available through an online database confirmed this trend at the protein level. The results for liver, breast, lung, and colon cancers were similar to those in previous research (11–13, 34). Takayama et al. (10) showed that high expression of USP10 is related significantly to poor prognosis in patients with prostate cancer, which is consistent with our experimental validation. However, Wang et al. (38) showed reduced expression of USP10 in human LUAD tissues, which contradicts the results of the present study, possibly because most of the samples analyzed in Wang’s study were derived from metastatic tumor tissues rather than *in situ* tumors. Furthermore, we found that *USP10* was overexpressed in gastric cancer, which is inconsistent with Zang et al.’s (7) findings and might be caused by the different subtypes of cancer and sample differences. Therefore, the sample size needs to be further expanded. Another exciting finding of this study was that the USP10 protein level was significantly lower in ovarian tumor tissues than in adjacent tissues. Furthermore, survival analysis found that low USP10 expression predicted poor prognosis in patients with ovarian cancer. A previous study by Han et al. (15) agreed with our findings and showed that differential *USP10* expression correlated with promoter hypermethylation. Whether USP10 has utility as an independent biomarker of prognosis in OV requires further biological experiments.

Kaplan–Meier survival analysis using the TCGA database demonstrated that in most cancer types (PAAD, LIHC, LUAD, and BRCA), high *USP10* expression was associated with poor prognosis. Similarly, as previously reported, *USP10* overexpression was shown to be associated with a shorter patient survival time (11, 12).. Moreover, *USP10* mutations are closely associated with the development of cancers. We postulated that *USP10* expression might be linked to the TMB in various cancers to influence patient survival and is a useful immunotherapy biomarker for checkpoint blockade selection in many types of cancer (39, 40). Analysis revealed that *USP10* expression was obviously associated with a high TMB in STAD, LUAD, and UCEC, which influenced patient OS. Previous studies have shown that the TMB predicted prognosis in patients with non-small-cell lung and colorectal cancers (41, 42). Further studies showed that high *USP10* expression correlated closely with the stage of cancer and the presence of lymph node metastasis in patients suffering from various types of cancer. These findings suggested that USP10 might serve as a predictable biomarker to determine the prognosis of different cancers. However, more in-depth molecular experimental evidence is needed to verify this.

Importantly, we found that in different types of cancer, *USP10* expression was associated with immune cell infiltration levels and has a critical function in cancer immunity, particularly in PAAD and LIHC. Thus, the results of the present study revealed the possible use of USP10 as a cancer biomarker and its function in tumor immunology. We revealed that the infiltration of dendritic cells, neutrophils, macrophages, CD4+ T cells, CD8+ T cells, and B cells in PAAD and LIHC are associated significantly with *USP10* expression; whereas, there was only a weak correlation between immune cell infiltration and *USP10* expression in LUAD. Interestingly, the association between the expression of *USP10* and the expression levels of marker genes of immune cells (e.g., *CD19*, *CD79A*, *CCL2*, *CD66b*, *HLA-DQB1*, *CD1C*, and *ITGAX*) was not always consistent with the overall trend, suggesting that specific interactions exist between USP10 and certain subtypes of immune cells. Furthermore, the close association between *USP10* expression and immune cell marker gene expression suggested that USP10 might function in PAAD and LIHC tumor immune regulation. DCs and macrophages, which are important antigen-presenting cells (APCs), were most related to the expression of *USP10* in LIHC. Tumor metastasis is promoted by DCs *via* their effects on Treg levels and the reduction in the CD8+ T cell response (43). However, in PAAD, DCs correlated weakly with USP10. These differences suggested heterogeneity between cancers that recruit APCs to the TME. A recent study showed that USP10 promotes tumor progression and TAM polarization in colorectal cancer (18). Therefore, our results revealed that USP10 might regulate TAM polarization.

In addition, Tregs are the most important cell type in the TME. Tregs are believed to suppress the excessive immune response by expressing cytotoxic T-lymphocyte associated protein 4 (CTLA4) and secreting IL-10 and transforming growth factor beta (TGFβ), thereby promoting the immune escape of tumor cells (44, 45). TGFβ signaling can be activated by USP10 depletion (46). Recently, researchers have found that depletion of Tregs does not prevent their suppressive activity. Moreover, the therapeutic effect of programmed cell death-1 (PD-1) and PD-1 ligand-1 (PD-L1) signaling blockade therapy on patients with tumors is still not as beneficial as expected. However, Maj et al. (47) showed that in the TME, Tregs are highly apoptotic and can greatly reduce the efficacy of PD-L1 anti-tumor immunotherapy. We further found a significant positive correlation between USP10 and genetic markers of Tregs (*CCR8* (encoding C-C motif chemokine receptor 8) and *STAT5B* (encoding signal transducer and activator of transcription 5B)) **(** **Table 1**), suggesting that USP10 could be involved in activating the immunosuppressive activity of Tregs in PAAD and LIHC. Moreover, in PAAD and LIHC, USP10 levels correlated significantly with several T helper cell markers (*STAT1*, *STAT6*, *STAT5A*, and *STAT3*) (**Table 1**). Studies have shown that STAT signaling is involved in numerous aspects of immune regulation, including immune escape and shaping the epigenetic structure of immune cells (48, 49). These findings suggest that USP10 might be closely related to STAT signaling to regulate tumor immune responses. Importantly, the prognosis of PAAD and LIHC was influenced by USP10 through immune cell infiltration. Taken together, USP10 is closely associated with immune cell activity in the TME and might affect patient prognosis through immune infiltration. These findings suggested that USP10 is an immune-related therapeutic target. Nevertheless, USP10’s exact role in tumor immunity requires further exploration.

In conclusion, we determined the universal applicability of USP10 in pan-cancer and found that high expression of USP10 is usually associated with poor clinical prognosis. Furthermore, USP10 is intimately linked to immune cell infiltration in certain cancers and might affect the overall survival of patients with PAAD and LIHC *via* immune infiltration. These results will enhance our understanding USP10’s vital function in tumorigenesis and serve as a useful basis for future studies.

## Data Availability Statement

The datasets presented in this study can be found in online repositories. The names of the repository/repositories and accession number(s) can be found in the article/**Supplementary Material**.

## Ethics Statement

The studies involving human participants were reviewed and approved by The Ethics Committee of Shanghai East Hospital Affiliated to Tongji University. Written informed consent for participation was not required for this study in accordance with the national legislation and the institutional requirements.

## Author Contributions

DG designed this study. DG, ZZ, RX, ZH, FL, YH, HC, JL, and XC collected the data and performed the bioinformatic analyses and visualization. DG, YH, and HC performed the hematoxylin and eosin staining and immunofluorescence staining experiments. YL and ZX provided study supervision and reviewed the manuscript. All authors contributed to the article and approved the submitted version.

## Funding

This work was supported by a grant from the National Natural Science Foundation of China [grant number 81972183].

## Conflict of Interest

The authors declare that the research was conducted in the absence of any commercial or financial relationships that could be construed as a potential conflict of interest.

## Publisher’s Note

All claims expressed in this article are solely those of the authors and do not necessarily represent those of their affiliated organizations, or those of the publisher, the editors and the reviewers. Any product that may be evaluated in this article, or claim that may be made by its manufacturer, is not guaranteed or endorsed by the publisher.

## Supplementary Material

Supplementary Figure 1 The protein level of USP10 in different types of cancer. Representative images of H&E stained normal and tumor slides (left 1st). Immunofluorescence staining analysis (left 2nd to 4th) showing an evident fluorescence signal in the tumor tissue sections compared with that in the control group. All scale bars = 50 μm. H&E, hematoxylin-eosin staining. N, normal tissues; T, tumor tissues.

Supplementary Figure 2 Protein expression of USP10 in cancers versus normal tissues as assessed in the HPA database. Compared to normal tissues, USP10 was significantly overexpressed in breast cancer, liver cancer, lung cancer, skin cancer, colon cancer, kidney cancer, and prostate cancer tissues. However, in ovarian cancer, there was a significant low expression of USP10 in tumor tissues compared with that in normal tissues. N, normal tissues; T, tumor tissues.

Supplementary Figure 3 TIMER database analysis of the correlation between *USP10* expression and immune cell infiltration levels in different cancer types. *USP10* expression levels in PAAD, LIHC, PCPG, PRAD, THCA, KIRC, and BLCA generally correlated positively with immune cell infiltration. However, in ACC, CHOL, HNSC-HPV+, UCEC, and UCS, *USP10* expression levels were not significantly correlated with infiltration of B cells, CD4+ T cells, CD8+ T cells, macrophages, neutrophils, and dendritic cells. Red box, positive Spearman’s correlation; blue box, negative Spearman’s correlation; gray box, no significant correlation. Significance is indicated by a *P*-value < 0.05.

Supplementary Figure 4 Analysis of the prognostic value of *USP10* expression in immune cell subsets in patients with PAAD and LIHC using Kaplan–Meier Plotter. **(A)** In PAAD, high *USP10* expression was linked to increased infiltration of B cells, NK cells, Tregs, and Th2 cells (all *P* < 0.05); and with decreased infiltration of CD4+ T cells, macrophages, and Th1 cells (all *P* < 0.05), and predicted inferior prognostic survival in patients with PAAD. However, *USP10* expression with increased (OS, HR = 2.41, *P* = 0.027) or decreased (OS, HR = 2, *P* = 0.012) CD8+ T cell infiltration had a marked impact on the survival of patients with PAAD and predicted a worse survival outcome. **(B)** In LIHC, overexpression of *USP10* and abundant infiltration of B cells and Treg cells (all *P* < 0.05) or reduced infiltration of Th1 cells (*P* < 0.05) predicted a worse prognosis. Interestingly, *USP10* expression in LIHC correlated statistically with enriched infiltration of macrophages (OS, HR = 1.76, *P* = 0.021), indicating a worse prognosis. In contrast, *USP10* expression was associated with reduced infiltration of macrophages (OS, HR = 0.54, *P* = 0.021), predicting a better survival outcome. PAAD, pancreatic adenocarcinoma; LIHC, liver hepatocellular carcinoma. OS, overall survival. HR, hazard ratio. Significance is indicated by a *P*-value < 0.05.
