# Supplementary material for: The Prognostic Value and Immune Infiltration of USP10 in Pan-Cancer: A Potential Therapeutic Target
Source: Front Oncol. 2022 Mar 31;12:829705. doi: 10.3389/fonc.2022.829705 (PMC9009419; doi:10.3389/fonc.2022.829705)
Supplement: Supplementary file 1 [file DataSheet_1.pdf]

Supplementary Materials

The Prognostic Value and Immune Infiltration of USP10 in Pan-Cancer: A Potential Therapeutic Target

Supplementary Table 1. Analysis of USP10 Expression in Cancer vs. Normal Tissue by Oncomine Database.

Cancer	Cancer Type	*P*-value	Fold Change	Rank (%)	Sample	Reference (PMID)
Blader	Superficial Bladder Cancer	1.91E-06	-1.613	12	157	16432078
Brain and CNS	Brain Glioblastoma	3.83E-06	-1.589	15	557	TCGA
Breast	Ductal Breast Carcinoma	9.86E-05	2.42	10	47	16473279
	Invasive Ductal Breast Carcinoma	7.45E-10	1.591	22	593	TCGA
Cervical	Cervical Cancer	2.52E-05	1.917	16	84	17510386
Colorectal	Cecum Adenocarcinoma	4.50E-11	1.755	4	237	TCGA
	Rectal Adenocarcinoma	1.27E-10	1.661	11	237	TCGA
	Colon Adenocarcinoma	6.57E-11	1.662	12	237	TCGA
	Colon Adenoma	1.85E-07	1.558	15	64	18171984
	Rectal Adenoma	3.45E-04	1.753	15	64	18171984
	Colon Carcinoma	2.55E-05	2.245	15	40	20957034
	Cecum Adenocarcinoma	5.09E-04	1.513	16	105	17615082
	Colorectal Carcinoma	7.44E-06	1.574	16	82	20143136
Gastric	Gastric Intestinal Type Adenocarcinoma	1.17E-05	1.755	16	69	19081245
Head and Neck	Head and Neck Squamous Cell Carcinoma	1.10E-08	1.862	5	54	14729608
	Floor of the Mouth Carcinoma	4.00E-04	2.025	8	84	17510386
	Tongue Carcinoma	1.18E-04	1.595	9	84	17510386
Kidney	Clear Cell Sarcoma of the Kidney	4.98E-06	-1.586	2	35	16299227
Leukemia	B-Cell Acute Lymphoblastic Leukemia	1.30E-04	1.659	3	28	16267031
Lung	Lung Adenocarcinoma	2.22E-08	1.559	7	156	20421987
	Squamous Cell Lung Carcinoma	5.33E-08	1.759	9	156	20421987
	Large Cell Lung Carcinoma	1.03E-04	1.637	12	156	20421987
Lymphoma	Anaplastic Large Cell Lymphoma	6.13E-05	1.848	10	60	17304354
	Angioimmunoblastic T-Cell Lymphoma	4.16E-04	1.851	15	60	17304354
Melanoma	Benign Melanocytic Skin Nevus	4.13E-05	2.252	4	70	16243793
	Cutaneous Melanoma	8.57E-06	3.033	6	70	16243793
Myeloma	Smoldering Myeloma	7.49E-04	1.584	28	78	17023574
Ovarian	Ovarian Carcinoma	1.77E-05	-1.669	15	195	18593951
Prostate	Prostate Carcinoma	8.32E-04	2.155	12	102	12086878
Sarcoma	Clear Cell Sarcoma of the Kidney	4.98E-06	-1.586	2	35	16299227
	Myxofibrosarcoma	8.41E-06	-1.503	7	158	20601955

Supplementary Table 2. Analysis of the Relationship between USP10 Expression and Progression in Different Cancer Patients by Prognoscan Database.

Cancer type	Dataset	Endpoint	N	Hazard ratio (95%)	Cox P
Bladder cancer	GSE5287	OS	30	1.94 [0.83 - 4.55]	0.126422
	GSE5287	OS	30	1.85 [0.63 - 5.47]	0.265252
	GSE13507	OS	165	0.89 [0.60 - 1.32]	0.557421
	GSE13507	DSS	165	1.21 [0.67 - 2.21]	0.525113
Blood cancer	GSE12417-GPL96	OS	163	0.92 [0.65 - 1.29]	0.627953
	GSE12417-GPL96	OS	163	0.97 [0.58 - 1.60]	0.898213
	GSE12417-GPL570	OS	79	1.24 [0.56 - 2.74]	0.594744
	GSE12417-GPL570	OS	79	1.18 [0.69 - 2.01]	0.541867
	GSE5122	OS	58	1.57 [0.91 - 2.71]	0.10791
	GSE5122	OS	58	1.64 [1.00 - 2.69]	0.052198
	GSE8970	OS	34	0.70 [0.37 - 1.31]	0.267191
	GSE8970	OS	34	1.25 [0.75 - 2.07]	0.386637
	GSE4475	OS	158	1.28 [0.70 - 2.33]	0.430043
	GSE4475	OS	158	1.61 [0.82 - 3.17]	0.165915
	E-TABM-346	OS	53	0.97 [0.29 - 3.28]	0.958764
	E-TABM-346	EFS	53	2.31 [0.82 - 6.50]	0.11104
	E-TABM-346	EFS	53	0.85 [0.27 - 2.71]	0.789643
	E-TABM-346	OS	53	2.42 [0.79 - 7.45]	0.123136
	GSE16131-GPL96	OS	180	0.70 [0.46 - 1.07]	0.096713
	GSE16131-GPL96	OS	180	0.62 [0.38 - 1.02]	0.061122
	GSE2658	DSS	559	1.66 [0.99 - 2.79]	0.056722
	GSE2658	DSS	559	1.52 [0.89 - 2.57]	0.121894
Brain cancer	GSE4271-GPL96	OS	77	0.47 [0.26 - 0.85]	0.012106
	GSE4271-GPL96	OS	77	0.58 [0.31 - 1.09]	0.092217
	GSE7696	OS	70	0.64 [0.16 - 2.56]	0.523636
	GSE7696	OS	70	1.02 [0.59 - 1.77]	0.930691
	MGH-glioma	OS	50	0.98 [0.36 - 2.66]	0.964566
	GSE4412-GPL96	OS	74	0.31 [0.16 - 0.59]	0.000393
	GSE4412-GPL96	OS	74	0.47 [0.22 - 1.04]	0.063053
	GSE16581	OS	67	20.90 [1.06 - 412.54]	0.04577
	GSE16581	OS	67	7.71 [0.52 - 113.28]	0.136261
Breast cancer	GSE19615	DMFS	115	1.90 [0.70 - 5.18]	0.209553
	GSE19615	DMFS	115	2.26 [0.65 - 7.85]	0.200771
	GSE3143	OS	158	1.43 [0.69 - 2.97]	0.338503
	GSE7849	DFS	76	0.84 [0.29 - 2.46]	0.748506
	GSE12276	RFS	204	1.28 [0.95 - 1.74]	0.109208
	GSE12276	RFS	204	1.19 [0.88 - 1.62]	0.255231
	GSE6532-GPL570	RFS	87	1.34 [0.63 - 2.86]	0.451899
	GSE6532-GPL570	DMFS	87	0.74 [0.33 - 1.63]	0.45023

GSE6532-GPL570	DMFS	87	1.34 [0.63 - 2.86]	0.451899
GSE6532-GPL570	RFS	87	0.74 [0.33 - 1.63]	0.45023
GSE9195	DMFS	77	1.78 [0.53 - 5.99]	0.350257
GSE9195	DMFS	77	1.81 [0.62 - 5.31]	0.280564
GSE9195	RFS	77	1.08 [0.38 - 3.07]	0.88166
GSE9195	RFS	77	1.46 [0.56 - 3.80]	0.435409
GSE12093	DMFS	136	0.54 [0.21 - 1.37]	0.19386
GSE12093	DMFS	136	0.86 [0.37 - 2.00]	0.721811
GSE11121	DMFS	200	0.89 [0.45 - 1.78]	0.749067
GSE11121	DMFS	200	3.51 [1.79 - 6.87]	0.000251
GSE1378	RFS	60	1.34 [0.83 - 2.19]	0.234268
GSE1378	RFS	60	1.56 [0.72 - 3.40]	0.259295
GSE1379	RFS	60	1.11 [0.56 - 2.24]	0.760776
GSE1379	RFS	60	0.90 [0.41 - 1.95]	0.789212
GSE9893	OS	155	1.56 [1.18 - 2.07]	0.001613
GSE2034	DMFS	286	0.74 [0.50 - 1.11]	0.144775
GSE2034	DMFS	286	1.36 [0.83 - 2.21]	0.223255
GSE1456-GPL96	OS	159	3.24 [1.50 - 7.02]	0.002777
GSE1456-GPL96	RFS	159	2.24 [1.19 - 4.23]	0.012635
GSE1456-GPL96	DSS	159	2.46 [1.18 - 5.14]	0.016407
GSE1456-GPL96	RFS	159	3.67 [1.73 - 7.77]	0.000677
GSE1456-GPL96	OS	159	1.87 [0.99 - 3.55]	0.053684
GSE1456-GPL96	DSS	159	3.78 [1.54 - 9.29]	0.003733
GSE7378	DFS	54	5.05 [1.61 - 15.87]	0.005601
GSE7378	DFS	54	1.63 [0.59 - 4.53]	0.347077
E-TABM-158	RFS	117	0.70 [0.33 - 1.45]	0.334027
E-TABM-158	DSS	117	0.35 [0.13 - 0.94]	0.036632
E-TABM-158	DMFS	117	0.52 [0.19 - 1.38]	0.186466
E-TABM-158	OS	117	0.70 [0.33 - 1.45]	0.334027
E-TABM-158	RFS	117	0.98 [0.55 - 1.74]	0.945936
E-TABM-158	DSS	117	0.78 [0.40 - 1.55]	0.482075
E-TABM-158	DMFS	117	1.04 [0.51 - 2.12]	0.922222
E-TABM-158	OS	117	0.98 [0.55 - 1.74]	0.945936
GSE3494-GPL96	DSS	236	1.52 [0.79 - 2.91]	0.206647
GSE3494-GPL96	DSS	236	1.56 [0.81 - 3.00]	0.183912
GSE4922-GPL96	DFS	249	1.67 [1.01 - 2.77]	0.045467
GSE4922-GPL96	DFS	249	1.85 [1.10 - 3.13]	0.020489
GSE2990	RFS	125	1.07 [0.54 - 2.13]	0.851484
GSE2990	DMFS	54	0.98 [0.53 - 1.83]	0.952689
GSE2990	RFS	62	1.02 [0.65 - 1.60]	0.932979
GSE2990	DMFS	125	0.73 [0.25 - 2.17]	0.570751
GSE2990	RFS	125	0.85 [0.37 - 1.95]	0.704236
GSE2990	RFS	62	1.03 [0.62 - 1.72]	0.901154

	GSE2990	DMFS	125	0.88 [0.34 - 2.23]	0.780581
	GSE2990	DMFS	54	0.99 [0.57 - 1.72]	0.970942
	GSE7390	RFS	198	1.33 [0.97 - 1.81]	0.072838
	GSE7390	DMFS	198	1.78 [1.15 - 2.74]	0.009123
	GSE7390	OS	198	1.63 [1.04 - 2.54]	0.031925
	GSE7390	DMFS	198	1.50 [1.04 - 2.17]	0.030162
	GSE7390	OS	198	1.50 [1.02 - 2.22]	0.041473
	GSE7390	RFS	198	1.51 [1.06 - 2.16]	0.023948
Colorectal cancer	GSE12945	OS	62	2.25 [0.45 - 11.28]	0.324629
	GSE12945	DFS	51	6.73 [0.49 - 93.36]	0.155194
	GSE12945	OS	62	2.79 [0.45 - 17.44]	0.27134
	GSE12945	DFS	51	4.51 [0.48 - 42.53]	0.18791
	GSE17536	DSS	177	0.79 [0.35 - 1.80]	0.574519
	GSE17536	OS	177	0.87 [0.50 - 1.49]	0.605519
	GSE17536	DSS	177	0.73 [0.40 - 1.36]	0.323454
	GSE17536	OS	177	1.26 [0.60 - 2.63]	0.536259
	GSE17536	DFS	145	0.81 [0.38 - 1.73]	0.59295
	GSE17536	DFS	145	0.52 [0.19 - 1.44]	0.208157
	GSE14333	DFS	226	0.46 [0.25 - 0.87]	0.016934
	GSE14333	DFS	226	0.86 [0.49 - 1.52]	0.607554
	GSE17537	OS	55	3.01 [1.29 - 7.04]	0.010908
	GSE17537	DFS	55	4.58 [1.90 - 11.03]	0.000694
	GSE17537	OS	55	2.70 [0.99 - 7.35]	0.05228
	GSE17537	DSS	49	4.09 [1.44 - 11.62]	0.0081
	GSE17537	DFS	55	3.06 [1.03 - 9.10]	0.044375
	GSE17537	DSS	49	2.10 [0.61 - 7.17]	0.237012
Esophagus cancer	GSE11595	OS	34	0.54 [0.18 - 1.65]	0.280266
	GSE11595	OS	34	0.93 [0.32 - 2.70]	0.889276
Eye cancer	GSE22138	DMFS	63	0.79 [0.45 - 1.41]	0.426063
	GSE22138	DMFS	63	0.67 [0.39 - 1.16]	0.14966
Head and neck cancer	GSE2837	RFS	28	0.60 [0.12 - 2.95]	0.531901
	GSE2837	RFS	28	2.84 [0.00 - 2016.13]	0.755772
Lung cancer	jacob-00182-CANDF	OS	82	0.44 [0.19 - 1.02]	0.054666
	jacob-00182-CANDF	OS	82	0.44 [0.12 - 1.62]	0.214745
	HARVARD-LC	OS	84	1.38 [0.77 - 2.46]	0.277518
	jacob-00182-HLM	OS	79	0.90 [0.38 - 2.12]	0.804682
	jacob-00182-HLM	OS	79	1.04 [0.39 - 2.76]	0.934357
	MICHIGAN-LC	OS	86	1.32 [0.43 - 4.04]	0.626447
	jacob-00182-MSK	OS	104	1.34 [0.29 - 6.26]	0.708144
	jacob-00182-MSK	OS	104	1.50 [0.57 - 3.93]	0.40888
	GSE13213	OS	117	2.55 [1.24 - 5.25]	0.011283
	GSE13213	OS	117	1.47 [1.05 - 2.06]	0.025945
	GSE31210	OS	204	3.66 [0.90 - 14.89]	0.070205

	GSE31210	RFS	204	5.58 [1.93 - 16.14]	0.001494
	GSE31210	OS	204	1.25 [0.30 - 5.12]	0.761312
	GSE31210	RFS	204	1.61 [0.56 - 4.63]	0.373167
	jacob-00182-UM	OS	178	0.82 [0.44 - 1.54]	0.53914
	jacob-00182-UM	OS	178	0.83 [0.33 - 2.06]	0.687809
	GSE11117	OS	41	2.19 [1.03 - 4.67]	0.041218
	GSE3141	OS	111	0.62 [0.34 - 1.16]	0.13556
	GSE3141	OS	111	0.66 [0.36 - 1.24]	0.196142
	GSE14814	OS	90	1.78 [0.69 - 4.64]	0.235101
	GSE14814	DSS	90	1.11 [0.38 - 3.26]	0.853846
	GSE14814	OS	90	1.28 [0.33 - 4.98]	0.721632
	GSE14814	DSS	90	0.82 [0.17 - 3.88]	0.806624
	GSE4716-GPL3694	OS	50	3.08 [0.44 - 21.50]	0.255716
	GSE8894	RFS	138	1.08 [0.86 - 1.36]	0.490194
	GSE8894	RFS	138	0.93 [0.64 - 1.35]	0.695719
	GSE4573	OS	129	1.23 [0.58 - 2.63]	0.584364
	GSE4573	OS	129	1.62 [0.87 - 3.03]	0.129342
	GSE17710	RFS	56	1.42 [0.68 - 2.95]	0.345448
	GSE17710	RFS	56	1.34 [0.64 - 2.81]	0.436522
	GSE17710	OS	56	1.56 [0.73 - 3.35]	0.249261
	GSE17710	OS	56	1.48 [0.69 - 3.18]	0.314743
Ovarian cancer	GSE9891	OS	278	0.64 [0.42 - 0.99]	0.043284
	GSE9891	OS	278	0.75 [0.56 - 1.00]	0.047007
	DUKE-OC	OS	133	1.14 [0.77 - 1.70]	0.510993
	DUKE-OC	OS	133	0.90 [0.67 - 1.22]	0.497325
	GSE8841	OS	81	0.54 [0.28 - 1.04]	0.066864
	GSE8841	OS	81	1.97 [0.70 - 5.55]	0.198476
	GSE26712	DFS	185	1.02 [0.70 - 1.50]	0.900119
	GSE26712	DFS	185	1.15 [0.83 - 1.58]	0.40071
	GSE26712	OS	185	0.93 [0.61 - 1.41]	0.722658
	GSE26712	OS	185	1.04 [0.73 - 1.49]	0.831497
	GSE17260	PFS	110	0.91 [0.54 - 1.56]	0.737392
	GSE17260	PFS	110	0.71 [0.43 - 1.17]	0.181207
	GSE17260	OS	110	1.36 [0.70 - 2.67]	0.36418
	GSE17260	OS	110	1.00 [0.52 - 1.91]	0.990622
	GSE14764	OS	80	1.75 [0.83 - 3.69]	0.138776
	GSE14764	OS	80	2.50 [1.01 - 6.20]	0.04842
Prostate cancer	GSE16560	OS	281	0.90 [0.62 - 1.32]	0.60319
Skin cancer	GSE19234	OS	38	6.18 [1.38 - 27.74]	0.017363
	GSE19234	OS	38	3.11 [0.93 - 10.38]	0.064624
Soft tissue cancer	GSE30929	DRFS	140	1.46 [0.65 - 3.26]	0.360254
	GSE30929	DRFS	140	0.91 [0.41 - 2.04]	0.824674
