# Supplementary figures and images for: The Prognostic Value and Immune Infiltration of USP10 in Pan-Cancer: A Potential Therapeutic Target
Source: Front Oncol. 2022 Mar 31;12:829705. doi: 10.3389/fonc.2022.829705 (PMC9009419; doi:10.3389/fonc.2022.829705)

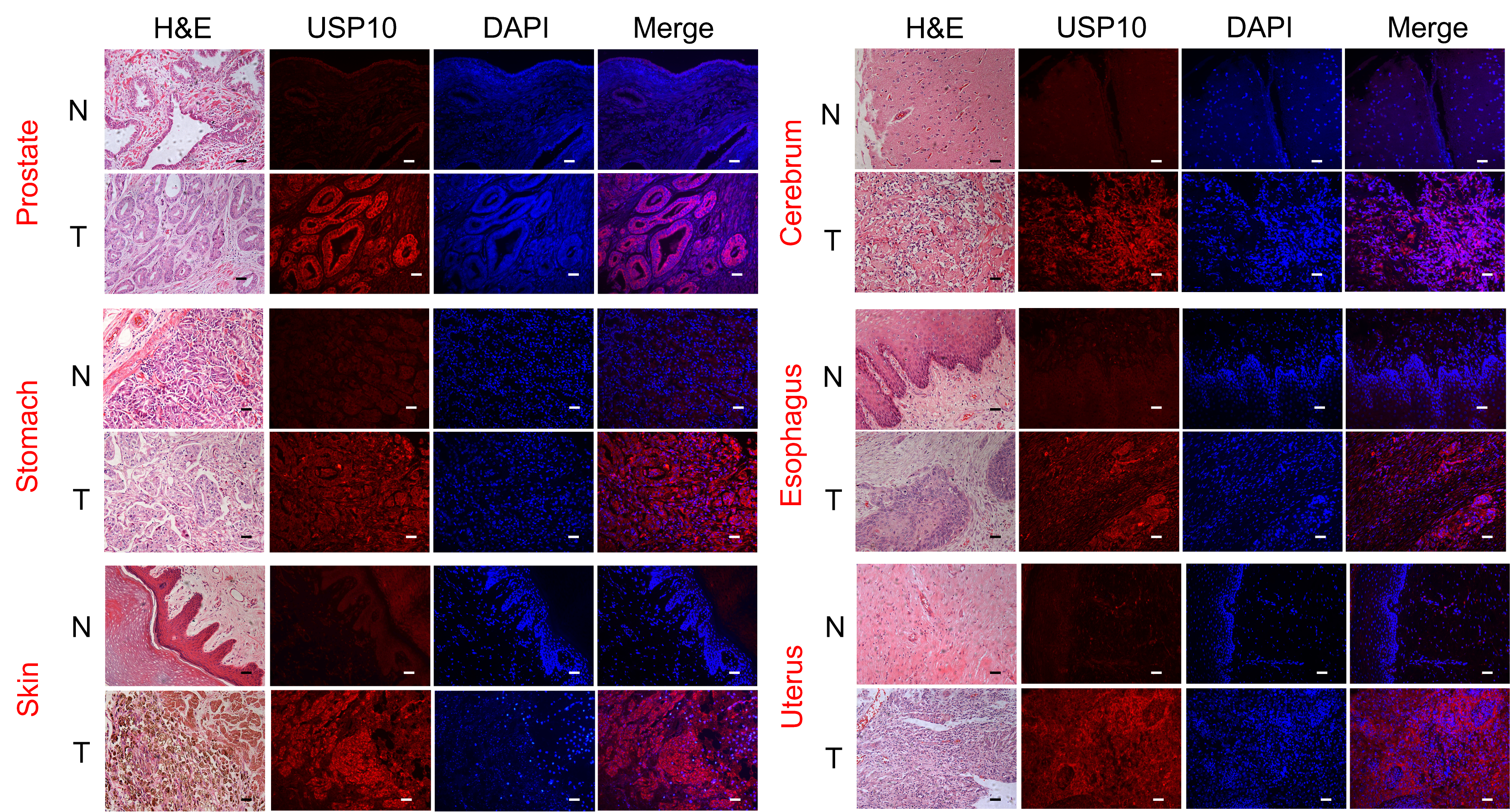

Supplement: Supplementary Figure 1 — The protein level of USP10 in different types of cancer. Representative images of H&E stained normal and tumor slides (left 1st). Immunofluorescence staining analysis (left 2nd to 4th) showing an evident fluorescence signal in the tumor tissue sections compared with that in the control group. All scale bars = 50 μm. H&E, hematoxylin-eosin staining. N, normal tissues; T, tumor tissues. [file Image_1.tif]

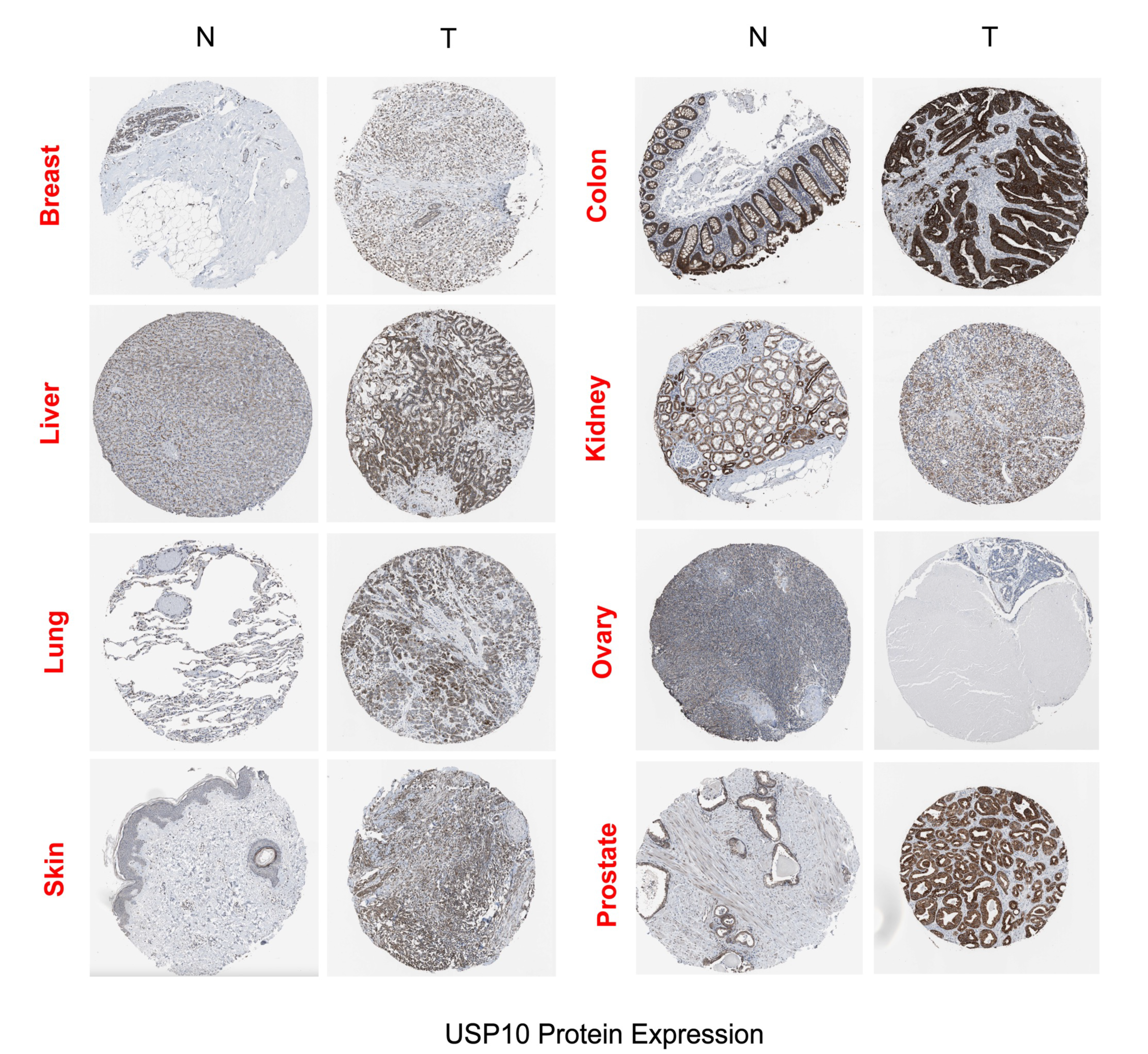

Supplement: Supplementary Figure 2 — Protein expression of USP10 in cancers versus normal tissues as assessed in the HPA database. Compared to normal tissues, USP10 was significantly overexpressed in breast cancer, liver cancer, lung cancer, skin cancer, colon cancer, kidney cancer, and prostate cancer tissues. However, in ovarian cancer, there was a significant low expression of USP10 in tumor tissues compared with that in normal tissues. N, normal tissues; T, tumor tissues. [file Image_2.tif]

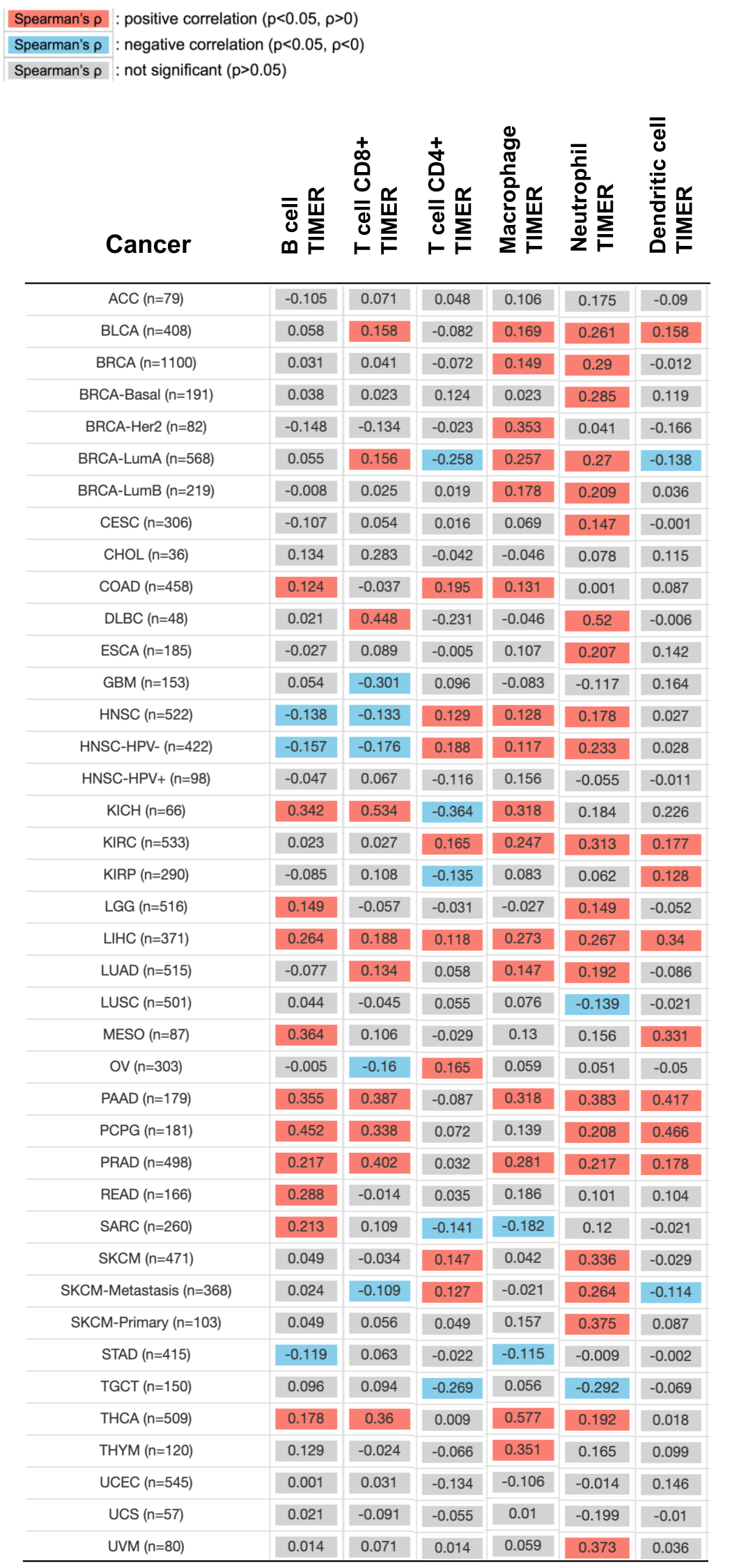

Supplement: Supplementary Figure 3 — TIMER database analysis of the correlation between USP10 expression and immune cell infiltration levels in different cancer types. USP10 expression levels in PAAD, LIHC, PCPG, PRAD, THCA, KIRC, and BLCA generally correlated positively with immune cell infiltration. However, in ACC, CHOL, HNSC-HPV+, UCEC, and UCS, USP10 expression levels were not significantly correlated with infiltration of B cells, CD4+ T cells, CD8+ T cells, macrophages, neutrophils, and dendritic cells. Red box, positive Spearman’s correlation; blue box, negative Spearman’s correlation; gray box, no significant correlation. Significance is indicated by a P-value < 0.05. [file Image_3.tif]

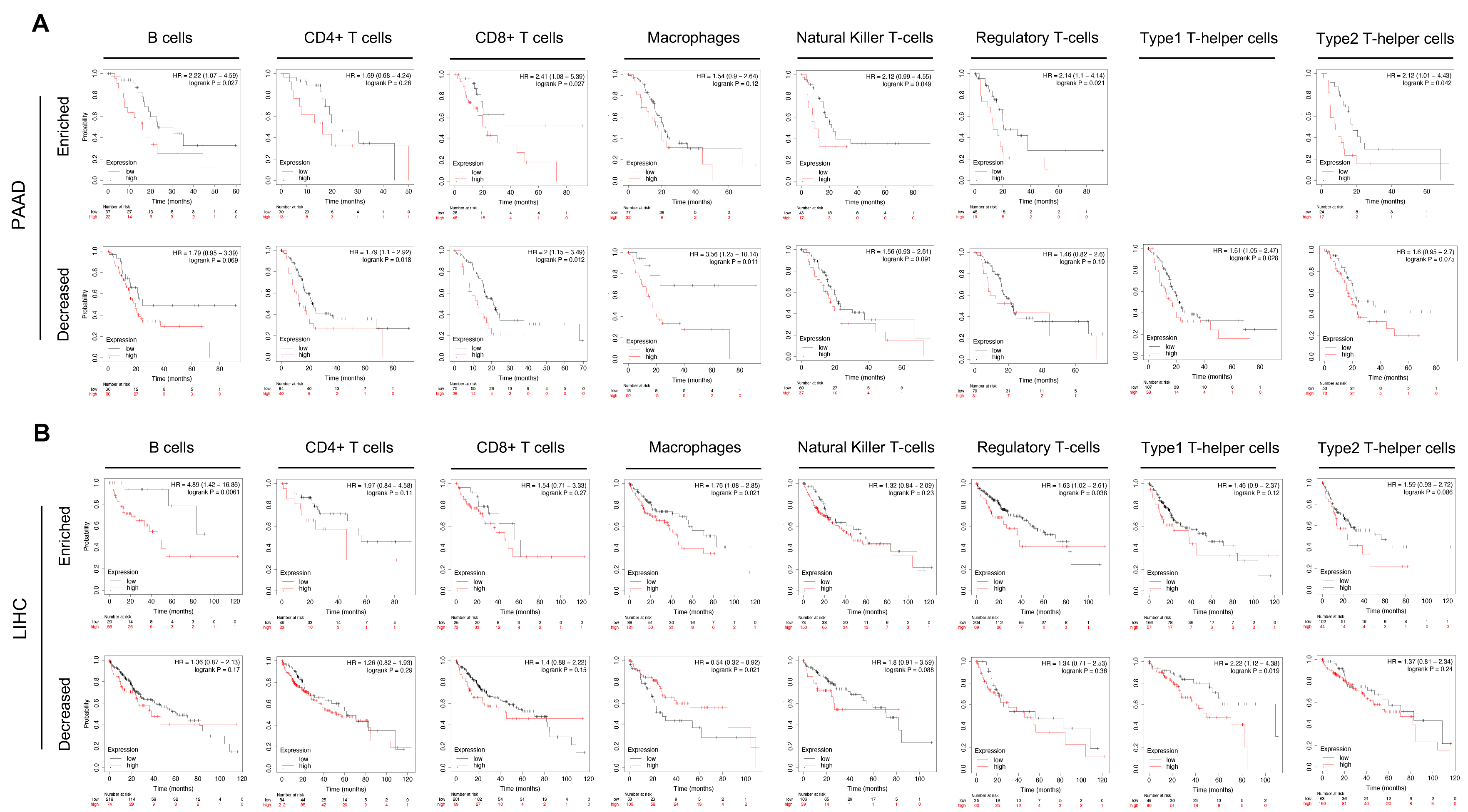

Supplement: Supplementary Figure 4 — Analysis of the prognostic value of USP10 expression in immune cell subsets in patients with PAAD and LIHC using Kaplan–Meier Plotter. (A) In PAAD, high USP10 expression was linked to increased infiltration of B cells, NK cells, Tregs, and Th2 cells (all P < 0.05); and with decreased infiltration of CD4+ T cells, macrophages, and Th1 cells (all P < 0.05), and predicted inferior prognostic survival in patients with PAAD. However, USP10 expression with increased (OS, HR = 2.41, P = 0.027) or decreased (OS, HR = 2, P = 0.012) CD8+ T cell infiltration had a marked impact on the survival of patients with PAAD and predicted a worse survival outcome. (B) In LIHC, overexpression of USP10 and abundant infiltration of B cells and Treg cells (all P < 0.05) or reduced infiltration of Th1 cells (P < 0.05) predicted a worse prognosis. Interestingly, USP10 expression in LIHC correlated statistically with enriched infiltration of macrophages (OS, HR = 1.76, P = 0.021), indicating a worse prognosis. In contrast, USP10 expression was associated with reduced infiltration of macrophages (OS, HR = 0.54, P = 0.021), predicting a better survival outcome. PAAD, pancreatic adenocarcinoma; LIHC, liver hepatocellular carcinoma. OS, overall survival. HR, hazard ratio. Significance is indicated by a P-value < 0.05. [file Image_4.tif]
